# From Seabed to Bedside: A Review on Promising Marine Anticancer Compounds

**DOI:** 10.3390/biom10020248

**Published:** 2020-02-06

**Authors:** Edina Wang, Maria Alba Sorolla, Priya Darshini Gopal Krishnan, Anabel Sorolla

**Affiliations:** 1 Harry Perkins Institute of Medical Research, QEII Medical Centre, Nedlands and Centre for Medical Research, The University of Western Australia, Crawley, WA 6009, Australia; 2 Biomedical Research Institute (IRB Lleida), Research Group of Cancer Biomarkers, 25198 Lleida, Spain

**Keywords:** marine extracts, anticancer agents, cancer models, clinical trials

## Abstract

The marine environment represents an outstanding source of antitumoral compounds and, at the same time, remains highly unexplored. Organisms living in the sea synthesize a wide variety of chemicals used as defense mechanisms. Interestingly, a large number of these compounds exert excellent antitumoral properties and have been developed as promising anticancer drugs that have later been approved or are currently under validation in clinical trials. However, due to the high need for these compounds, new methodologies ensuring its sustainable supply are required. Also, optimization of marine bioactives is an important step for their success in the clinical setting. Such optimization involves chemical modifications to improve their half-life in circulation, potency and tumor selectivity. In this review, we outline the most promising marine bioactives that have been investigated in cancer models and/or tested in patients as anticancer agents. Moreover, we describe the current state of development of anticancer marine compounds and discuss their therapeutic limitations as well as different strategies used to overcome these limitations. The search for new marine antitumoral agents together with novel identification and chemical engineering approaches open the door for novel, more specific and efficient therapeutic agents for cancer treatment.

## 1. Introduction

Marine ecosystems are composed of a complex community of interacting organisms, including bacteria, protozoans, algae, chromists, plants, fungi, and animals. They live confined within an aquatic saline environment that covers 71% of the earth’s surface and accounts for 90% of the earth’s biosphere. The biodiversity of marine ecosystems is extremely rich and is considered a virtually unlimited source of bioactive compounds [1]. In addition, the marine environment is characterized for being extremely harsh and exposed to life-threatening conditions such as lack of light, lack of nutrients, extreme pH and pressure, highly variable weather conditions, and predator attacks. For this reason, marine organisms have undertaken adaptive mechanisms and symbiotic interactions, among others, that translate into unexpected biochemical pathways leading to an astonishingly wide range of metabolites, secondary metabolites, and toxins [2].

This “marine wealth” in bioactives has attracted different types of industries, including pharmaceutical, cosmetic, nutraceutical, and agrochemical businesses [3,4]. With regards to pharmaceutical activity, Big Pharma has raised its interest in the exploitation of the marine environment, driven by the broad range of bioactivities that the sea ecosystem offers such as anticancer, anti-inflammatory, antibacterial, antiviral, antifungal, antifouling, antiprotozoal, anticoagulant, immunosuppressive, and neuroprotective activities [5]. Furthermore, marine bioactives display higher cytotoxicity compared to terrestrial natural products, which stresses their advantage in terms of potency as anticancer agents. Interestingly, there are more agents with antitumor properties in the same number of marine extracts than in terrestrial extracts. A preclinical cytotoxic screen carried out by the U.S. National Cancer Institute concluded that ~1% of the marine samples analyzed possessed antitumoral activity while the same was true for the ~0.2% of terrestrial samples tested [6]. Of note, the phylum *Porifera* (sponges) was the marine organism group that displayed a higher percentage of strong cytotoxic bioactives (IC_50_ <2 µg/mL), followed by the phylum *Bryozoa*, *Chordata*, and *Cnidaria* [6,7]. Undoubtedly, the immense marine biodiversity, comprised of ~230,000 known species, combined with their associated bioactives, represents an immense reservoir of anticancer agents, the market value of which is believed to range between USD 563 billion and USD 5.69 trillion [8]. The same environmental–economic report predicted the existence of 253,120 to 594,232 novel anticancer chemicals in marine organisms and that between 90.4% and 92.6% of these compounds are yet to be discovered [8]. This data not only highlights the lack of exploration of the marine environment but also the potential therapeutic significance that it holds as a source of anticancer therapeutics.

The first exploratory journey on the search of marine bioactives was initiated by Bergmann in the 1950s. Bergmann et al. reported the first discovery of two bioactive nucleosides, spongouridine and spongothymidine, extracted from the sponge *Cryptotethia crypta* [9]. These nucleosides represented the starting point for the synthesis of Ara-A and Ara-C (or Cytarabine). Importantly, Cytarabine has been the cornerstone treatment for acute myelogenous leukemia for more than thirty years [10,11]. Currently, there are eight anticancer drugs approved by the US Food and Drug Administration (FDA), the European Evaluation Medicines Agency (EMEA), or the Australian Therapeutic Goods Administration (ATGA) of marine origin, plus a few in phase I, II or III clinical pipelines [12]. Interestingly, aside from Cytarabine, all other anticancer drugs of marine origin have been approved in the last twenty years [12], anticipating that the years to come will be especially prolific for marine anticancer drug discovery. Indeed, it has been predicted that between 55 to 214 new marine anticancer drugs will advance for cancer treatment in the clinic [8], given the large marine biodiversity that is yet to be uncovered. However, the ecological impact of human activities and the intrinsic limitations of the marine ecosystem can certainly decrease those numbers. Apart from the continued degradation of marine habitats, there is a range of limitations that can hamper the clinical development of marine-derived drugs such as lack of sustainable supply, low production, structural complexity, phenotypic variations, moderate efficiency, and poor antitumor effectivity and selectivity [12]. However, there are ongoing strategies than can aid in overcoming the limitations presented and accelerate their translation into the clinic.

In this review, we outline highly potent and promising antitumoral compounds isolated from marine organisms, in particular, marine flora and invertebrate fauna. We also focus our manuscript on studies that have investigated anticancer activity in relevant in vivo cancer models and/or those that successfully inhibit tumor cell proliferation in the nanomolar or low micromolar range (Table 1, Table 2, Table 3, Table 4, Table 5, Table 6 and Table 7), as these reports can better validate the antitumoral activity of marine products and their applicability for future cancer therapy in humans. We have also listed the anticancer drugs with marine origin that have been institutionally approved together with those under current evaluation in clinical trials. Lastly, we have identified current limitations for the clinical development of marine compounds and strategies being adopted to overcome these limitations.

## 2. Antitumoral Compounds Originated from Marine Flora

Marine flora refers to bacteria, actinobacteria, cyanobacteria, and fungi (also known as microflora), and microalgae, macroalgae, mangroves, and other higher plants living in a marine environment. Of note, microflora and microalgae alone account for more than 90% of the oceanic biomass [13]. Thus, marine flora is considered one of the richest sources of antitumoral drug candidates on Earth. However, due to the lack of medical focus and efficient extraction technologies, the real impact that marine flora could have for the development of anticancer drugs is relatively unknown compared to terrestrial flora [14]. Nevertheless, multiple studies have reported antitumoral properties of chemicals extracted from this group of organisms.

### 2.1. Bacteria, Actinobacteria, and Cyanobacteria

Microorganisms in these groups produce toxins and metabolites that possess anticancer activity. Interestingly, as a consequence of the association with marine bacteria, a high percentage of molecules from sea animals and algae displays antitumoral properties [15]. Examples are the depsipeptide Kahalalide F from the bacterial symbiont *Vibrio mediterranei* that interacts with the marine mollusk *Elysia rufescens* or Dolastatin 10 (Figure 2), a pentapeptide from the cyanobacteria *Symploca sp.* which preys the sea hare *Dolabella auricularia* (discussed in Mollusks). A Dolastatin 10 analog, the linear pentapeptide Symplostatin 1, isolated from the cyanobacteria *symploca sp.* showed potent inhibition of cell proliferation in vitro with IC_50_ in the subnanomolar range in LoVo and KB cell lines. In vivo, Symplostatin 1 suppressed the growth of the murine colon adenocarcinoma 38 and the murine mammary adenocarcinoma 16/C when mice were administered intravenously (i.v.) with 0.5 and 0.25 mg/Kg of extracts [16,17]. Another report displayed strong potency in vitro of Symplostatin 1 in both cancer cell lines MDA-MB-435 (melanoma) and SK-OV-3 (ovarian) and normal cells (HUVEC) [17]. Another Dolastatin derivative, a synthetic derivative of Dolastatin 10, the tetrapeptide TZT-1027, demonstrated strong antitumoral effects in murine P338 leukemia, B16 melanoma, colon 26 adenocarcinoma, and M5076 sarcoma models in mice treated intraperitoneally (i.p.) and i.v., with low mg/Kg of extracts [18]. In 2008, TZT-1027 was assessed in non-small cell lung cancer (NSCLC) patients in a phase I clinical trial [19]. However, results were not astonishing, only observing one complete response and three partial responses in the 49 patients evaluated [19].

Pyrroloformamide is another example of bioactives coming from marine bacteria interacting with a superior organism. Pyrroloformamide is produced by the actinobacteria *Streptomyces sp.*, which lives in symbiosis with the ascidian *Eudistoma vannamei*. This bioactive successfully prevented cell division in the metastatic prostate cancer cell line PC3M presenting an IC_50_ of 1.67 µM [20]. Another promising antitumoral compound extracted from *Streptomyces sp.* is Cromomycin A2. This aureolic acid promoted autophagy in the metastatic melanoma cell line MALME-3M and presented an IC_50_ of 16.7 nM [21]. Other potent cytotoxics derived from bacteria are Anthracyclinones 1 and 4 extracted from *Micromonospora sp.* These bioactives induced cytotoxicity in the human colon adenocarcinoma cell line HCT-8 within the low micromolar range [22]. Coibamides are another group of chemicals that is appealing for anticancer treatment, in particular, Coibamide-A from the cyanobacteria *Leptolyngbya sp.* This cyclic depsipeptide exhibited cytotoxic effects in NCI-H460 lung cancer cells and mouse neuro-2a cells in the nanomolar range, among other cell lines [23].

Karpinski et al. have reviewed numerous peptides belonging to the non-ribosomal peptide class and present in marine bacteria which are found in marine sediments of different depths that have exhibited potent anticancer activity in vitro [24]. Among these active peptides, those presenting IC_50_ in the nanomolar range are the most compelling. Peptides such as Lucentamycins A from the actinobacteria *Nocardiopsis lucentensis* and Mixirins A, B, and C from *Bacillus sp.,* with activity against the human colon carcinoma cell line HCT-116 [25,26]; Ohmyungsamycins A and B from actinobacteria *Streptomyces sp*. [27], which displayed antiproliferative effects in several human cancer cell lines A519 (lung), SNU-638 (stomach), MDA-MB-231 (breast), and SK-HEP-1 (liver); and Urukthapelstatin A from actinobacteria *Mechercharimyces asporophorigenens,* with observed anticancer activity in the human lung cancer lines A549, DMS114, and NCIH460, human ovarian cancer cell lines OVCAR-3, OVCAR-4, OVCAR-5, OVCAR-8, and SK-OV3, human breast cancer cell line MCF-7 and in the human colon cancer cell line HCT-116 [28,29]. Interestingly, these peptides are not synthesized by ribosomes but other equivalent prokaryotic complexes and contain D-amino acid residues and other structural peculiarities rarely found in nature [24].

Although chemicals from marine bacteria have shown successful inhibition of tumor growth in many in vitro and in vivo cancer models and even some discrete efficacy in patients, none of the compounds have been approved institutionally for routine use on patients.

### 2.2. Fungi

Marine fungi can be found freely in water or sea sediments and are also associated with, for example, microalgae, macroalgae, mangroves, sponges, mollusks, and crustaceous. It is believed that fungi synthesize totally different biologically active compounds depending on their living ecological, physical, and biological conditions [30]. In fact, sponges, algae, and mangroves benefit from fungi and their cytotoxic metabolites to survive in the extreme oceanic environment [31]. Marine endophytic fungi (or fungi internally living in plants) are the most potent antitumoral compounds among marine fungi. One example of compounds with strong anticancer activity (in the nanomolar range) is compound *4*, the disulfide-bridged diketopiperazine brocazine G, from the mangrove-derived endophytic fungus *Penicillium brocae* MA-231. This compound induced cytotoxicity in the cisplatin-sensitive and cisplatin-resistant human ovarian cancer cell lines A2780 [32]. Another example is compound 2, a sesquiterpenoid from the endophytic fungi *Penicillium sp. FJ-1* associated with the mangrove *Avicennia marina*. This metabolite triggered significant DNA fragmentation (apoptosis) in the human osteosarcoma cell line MG-63. Also, this compound inhibited in vivo tumor growth of MG-63 xenografts when administered intragastrically six times per week at 10 and 30 mg/Kg [33]. Halimide (Figure 2) is perhaps the most clinically successful metabolite from marine fungi. Halimide was originally isolated from *Aspergillus sp.* living in seaweeds in Phillippines waters and is a diketopiperazine alkaloid. Synthetically synthesized as Plinabulin (NPI2358), this dehydrodiketopiperazine derivative of Halimide has been tested in early phase clinical trials, showing promising anticancer responses [34,35]. Moreover, Plinabulin is currently being evaluated in a phase III clinical trial (DUBLIN-3) in combination with docetaxel (NCT02504489), and in a phase I/II trial in combination with nivolumab (NCT02812667) in patients affected by advanced NSCLC. Plinabulin is categorized as a tubulin polymerization inhibitor interacting with the colchicine-binding domain of β-tubulin [36].

### 2.3. Microalgae

Microalgae, together with cyanobacteria, represents the major component of marine phytoplankton comprising more than 30,000 species [37]. Microalgae are eukaryotic unicellular organisms that possess a large variety of pigments, lipids, carotenoids, omega-3 fatty acids, polysaccharides, and vitamins that have attracted great interest from the cosmetic, pharmaceutical and food industries [38]. Phytochemicals from microalgae are an interesting source of anticancer compounds and possess more potent biological activities than the ones present in terrestrial plants. Similar to fungi, microalgae have the ability to grow alone or in association with other marine organisms and they can adapt to extreme adverse environments by producing bioactives essential for survival and defence. Taxonomically, microalgae are classified into four main groups: red, brown, blue-green (cyanobacteria), and green microalgae, according to their natural color. Multiple microalgae compounds show prominent anticancer activity in vivo, thus presenting a promising clinical potential. One such compound is Astaxanthin. Astaxanthin is a keto-carotenoid originated from the green microalgae *Haematococcus pluvialis*. This compound has shown anti-proliferative effects in a chemically-induced model of colon carcinogenesis in rats [39]. Another one is the extracts from the green microalgae *Chlorella sorokiniana*. The water reconstituted powders of this algae significantly reduced in vitro tumor growth in the human lung adenocarcinoma cell lines A549 and CL1–5 [40] by at least 40% when treated at 250 ng/mL and induced apoptosis. Notably, CL1-5 xenografts implanted in nude mice experimented significant shrinkage [40] when mice were fed daily with 50 mg/Kg of *Chlorella*’s extracts.

### 2.4. Macroalgae

Macroalgae, also denominated as algae or seaweeds, comprise 30,000 species and are eukaryotic, multicellular organisms. According to their natural pigmentation, macroalgae are classified in brown algae (Phaeophyceae), red algae (Rodophyta), and green algae (Clorophyta). Many macroalgae metabolites have been acknowledged as compounds with excellent nutritional and pharmaceutical properties. Of note, sulfated polysaccharides, carotenoids, and phlorotannins from marine macroalgae have been proposed as excellent adjuvant supplements for cancer management or used directly for cancer treatment [41]. In fact, most of the in vivo studies assessing the anticancer potential of macroalgae compounds have focused on sulfated polysaccharides.

One such prominent compound would be the sulfated polysaccharide H3-a1. This polysaccharide was isolated from the brown seaweed *Hydroclathrus clathratus* and showed inhibition of in vivo tumor growth of murine ascitic Sarcoma 180 and prolonged mice life span by 30%–40% [42]. Also, the alkali-extracted polysaccharide DAEB from the green algae *Enteromorpha intestinalis* exhibited a reduction of tumor growth in the same cancer model when mice were fed for ten days with DAEB. The mechanism of tumor reduction was presumably due to the immune-stimulatory properties of the compound through TNF-α induction [43]. In addition, the Grateloupia longifolia polysaccharide (GLP) from the red algae *Grateloupia longifolia* reduced in vivo tumor growth in the same model by 52% and also tumor-associated vascularization by 64% when animals were administered i.v. with 200 mg/Kg of GLP [44]. Another sulfated polysaccharide from a red algae, *Eucheuma serra agglutinin* (ESA), significantly inhibited tumor growth of Colon26 tumors allografts when mice were treated i.v. every three days with 400 µg of ESA [45]. Moreover, the polysaccharide SargA from the brown algae *Sargassum stenophyllum* substantially reduced in vivo tumor growth of B16F10 melanoma tumors when administered subcutaneously daily for three days near to the tumors at doses of 1.5 or 150 µg/animal [46]. This same sugar was also able to disrupt angiogenesis. Furthermore, the marine-derived sulfated polysaccharide (MSP) from a brown algae successfully suppressed the formation of metastasis in vivo in a model of Lewis lung carcinoma when mice were injected i.p. with MSP at 40 mg/Kg [47]. Another polysaccharide with anti-metastatic potential is calcium spirulan from *Spirulina platensis*. This compound reduced the growth of the spontaneous melanoma lung metastasis model B16-BL6 when mice were administered i.v. seven times with 100 µg of the sulfated sugar [48].

Most probably, Fucodian would be the most therapeutically promising sulfated polysaccharide (Figure 2). Fucoidan is a compound found on the cell wall of many species of brown macroalgae that has been evaluated in vitro and in vivo in a wide range of cancers, including breast cancer [49], head and neck cancer [50], and lung cancer [51]. In humans, Fucoidan has been prescribed as a food supplement to reduce the secondary effects derived from chemotherapy, such as fatigue [52], or to enhance efficacy of chemotherapy. A double-blind randomized clinical trial performed in Taiwan showed an increase in disease control in patients affected by metastatic colorectal cancer when treated with folinic acid, 5-fluorouracil, and irinotecan plus bevacizumab therapy [53]. Currently, there is an ongoing phase II clinical trial (NCT04066660) in 100 patients with advanced hepatocellular carcinoma, assessing the antitumoral effect of the dietary consumption of Fucoidan, with results yet to be reported. Despite evidence of the anticancer activity in vitro and in vivo of Fucoidan and its benefits in combination with standard therapy in humans, it is probably too early to draw solid conclusions on its therapeutic advantage. One of the reasons is the highly complex chemical structure of Fucoidan, which varies depending on its biological origin. These differences are suggested to greatly affect its potency [54].

### 2.5. Mangroves and Other Higher Plants

Similar to other marine organisms, marine plants have been largely unexplored for new antitumoral bioactives in comparison to their terrestrial counterparts. The primary reason for the lack of studies would be due to the inaccessibility [55]. Marine plants are adapted to live in harsh and changing environments and are the source of bioactives with unique and yet-to-know anticancer properties. One of the most highly distinguished plants in terms of producing anticancer chemicals is the mangrove. Mangroves are a highly unexplored group of marine plants found mostly in tropical zones with high salinity and temperature, anaerobic soils, and rapidly changing weather conditions [55]. To be able to adapt to this extreme environment, mangroves produce a wide range of bioactive compounds including hormones, primary and secondary metabolites, and antioxidants. Several compounds isolated from mangroves have been highlighted for its anticancer activity in vivo. For example, Chakraborty et al. demonstrated that extracts from *Acanthus ilicifolius Linn*. administered daily through i.p. at 2.5 mg/Kg to mice previously i.p. injected with Ehrlich ascites carcinoma cells reduced tumor cells counts in their ascitic fluid and extended their life span [56]. Also, Chu et al. reported significant in vivo anti-proliferative and anti-metastatic effects in mice bearing subcutaneous Lewis lung carcinoma tumors after being fed with leaf extracts of *Terminalia catappa* L. at the dose of 3 g/day/Kg. In particular, the extracts decreased tumor growth of primary tumors and the percentage of metastasis by 68% without evident signs of toxicity [57]. In addition, Neumann et al. showed that Tagalsin C (TC), a dolabrane-type of diterpene isolated from *Ceriops tagal,* reduced in vivo tumor growth of human T cell leukemia xenografts (CEM cells) when administered i.p. at the dose of 50 mg/Kg, the first week daily, and the other, three times per week. Notably, no tumor developed in two mice (out of seven) and only a slight change in body weight was recorded [58]. Moreover, Jones et al. described an in vivo antitumoral effect for 3-chlorodeoxylapachol, a naphthoquinone isolated from leaves and twigs of the black mangrove *Avicennia germinans*. This compound was administered i.p. in mice bearing fibers filled with KB (oral cancer), LNCaP (prostate cancer), and hTERT-RPE1 (pigment epithelial) cells inserted into the peritoneal cavity and under the skin. 3-Chlorodeoxylapachol at the dose of 5 mg/Kg displayed significant in vivo reduction of the engineered KB tumors [59]. Furthermore, Prabhu et al. reported an antitumoral activity for *Rhyzophora apiculata* extracts in the melanoma mice model B16F10 when mice were treated daily with 10 mg/Kg of mangrove extracts during ten days. Mice tumors reduced in volume by approximately one-third and life span increased by 50% in the animals [60].

## 3. Antitumoral Compounds Originated from Marine Invertebrate Fauna

**Figure 1:**
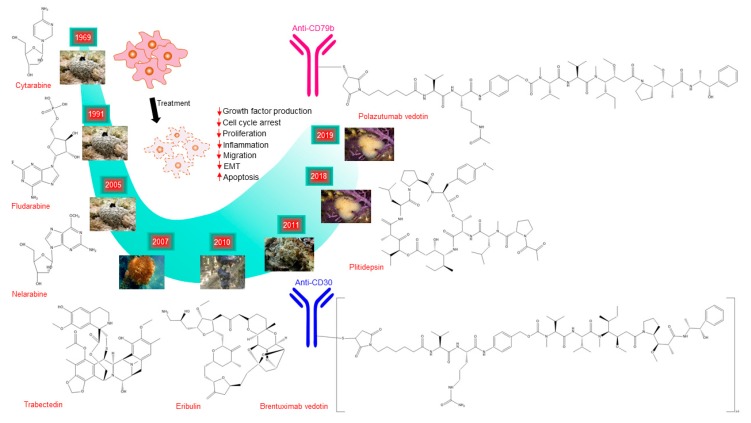
Schematic representation of the discovery timeline of marine-derived drugs approved for cancer treatment. It shows their chemical structures, images of the marine organism where these drugs were first originated and the major biological effects of the drugs on cancer cells. The images for Cytarabine, Fludarabin, Nelarabine, and Eribulin are adapted from http://spongeguide.org. The images for Trabectedin and Brentuximab vedotin are adapted from http://bioweb.uwlax.edu and http://seaslugs.free.fr, respectively. The images for Plitidepsin and Polazutumab vetodin are from PharmaMar.

Strikingly, more than 50% of the FDA-approved drugs during the 1980s and the 1990s are derived from marine life. Additionally, most of these marine pharmaceutical drugs have originated in marine invertebrates that include sponges, tunicates, mollusks, and bryozoans. Importantly, eight out of ten marine-derived drugs approved for cancer treatment came from marine invertebrates [12,97] (Figure 1 and Table 8). Thus, invertebrate fauna, in particular sponges, tunicates, mollusks, and bryozoans, is currently the main source of anticancer drugs. For this reason, we have focused our review on those four types of invertebrate animals.

### 3.1. Sponges

**Figure 2:**
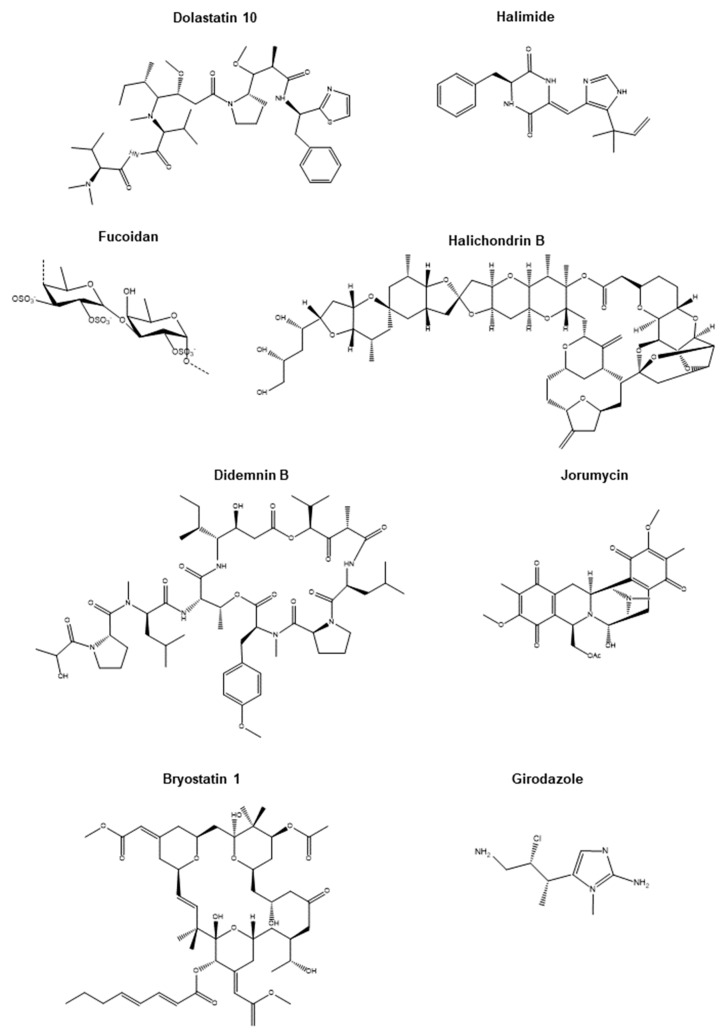
Chemical structures of promising marine-derived anticancer compounds. Chemical structures of Dolastatin 10, Halimide, Fucoidan, Halichondrin B, Didemnin B, Jorumycin, Bryostatin 1, and Girodazole.

Sponges, which belong to the phylum *Porifera*, are sessile organisms that totally rely on secreted toxins and secondary metabolites for their self-defense against predators. This makes these organisms one of the most productive sources of bioactives, including therapeutic agents for cancer. The journey for the search of novel sponge bioactives began in the 1950s by Bergmann and coworkers with the isolation of the nucleosides, spongouridine and spongothymidine, from the marine sponge *Cryptotethya crypta* [9]. Notably, Spongohymidine represented the basis for the synthesis of Ara-C (also known as Cytarabine), the first marine-derived anticancer agent. Cytarabine was approved in 1969 by the FDA and marketed under the trade name of Cytosar-U. Currently, Cytarabine is mainly used for the treatment of acute leukemia and lymphoma. Another sponge compound whereby its synthetic version has reached clinical trials is Halichondrin B (Figure 2). Halichondrin B is a polyether macrolide originally isolated from the marine sponge *Halichondria okadai* in 1986 by Hirata et al. [61]. In their report, they demonstrated potent in vivo anticancer activity of Halichondrin B against murine melanoma B16 tumors and P338 and L-1210 leukemias in mice receiving the compound i.p. or i.v. at doses ranging from 1.25 to 100 µg/Kg. Moreover, the in vivo antitumoral properties of Halichondrin B captured the interest of the U.S. National Cancer Institute for the development of this novel chemotherapeutic drug. However, due to the lack of sustainable supply, the development of Halichondrin was haltered. Fortunately, in 1992, Aicher et al. successfully attempted the synthesis of Halichondrin in the laboratory [98] and it was also discovered that the activity of Halichondrin B lies in the macrocyclic C1-C38 moiety. Altogether, this settled the basis for the development of C1–C38 analogs. Among them, Eribulin (also known as R-086526 or NSC-707389) deserves special mention as it showed in vivo antitumoral efficacy in multiple xenografts through microtubule destabilization [62] and successful increase of overall survival in the phase III clinical trial EMBRACE in patients with heavily pretreated metastatic breast cancer [99]. This culminated in its approval in 2010 by the FDA for the treatment of those metastatic breast cancers previously treated with chemotherapy regimens. Eribulin is marketed by Eisai Co. under the trade name Halaven.

An initially promising anticancer compound extracted from sponges is Girodazole (Figure 2). Girodazole, which possesses an atypical chemical structure compared to other anticancer agents, was isolated from the sponge *Pseudaxinyssa cantharella* in 1991 and found to have anticancer activity in vivo in leukemia, mammary adenocarcinoma, and histiocytosarcoma allografts without observed toxicity in mice and dogs [63]. However, posterior testing in humans was discontinued due to its low activity at the highest tolerated dose of 15 mg/m^2^ [100]. Agelasphin-11 is another promising anticancer agent. This galactosylceramide isolated from extracts from the sponge *Agelas mauritianus* prolonged the life span of mice intraperitoneally injected with murine melanoma B16 cells, presumably through an immunogenic response triggered by natural killer cells. However, no tumor growth reduction was observed in the mice [64]. Another sponge chemical showing in vivo antitumoral effects is Pachymatismin. This compound is a glycoprotein extracted from the marine sponge *Pachymatisma johnstonii* that significantly reduced the growth of human NSCLC xenografts (N6 cells) after three weeks of treatment [65]. Also, the marine sponge alkaloid Naamidine, isolated from *Fijian Leucetta sp.,* significantly decreased the growth of A431 squamous cell carcinoma xenografts (EGF responsive) by 85% when mice were treated daily for five days at the maximum tolerated dose of 25 mg/Kg, and more strikingly by 92.5% with 50 mg/Kg. However, a higher dose resulted in high mice mortality (two out of six mice) [66]. The mechanism of action of Naamidine was through the inhibition of EGF-mediated DNA synthesis and cell proliferation but not through disturbing EGF–EGFR binding [66]. This same year, Tsuchiya et al. isolated Scalarane sesterterpenes 1–4 from extracts of the sponge *Hyrtios erecta*. Compound *1* was the most potent, inducing cytotoxicity in vitro at doses ranging from 14.5 to 57.7 ng/mL in the mouse lymphatic leukemia cell line P338 and in the human gastric cancer cell lines MNK-1, MNK-7, and MNK-74. In vivo, animals inoculated i.p. with P388 cells and injected i.p.with 0.5 to 8 mg/Kg of compound *1* on days 1, 5 and 9 after P388 cells injection lengthened their life span by 24%–74% [67]. Another sponge compound that demonstrated potent in vivo anticancer activity is B6. B6 is a bromopyrrole isolated from extracts of the marine sponge *Polymistia sp*. that was shown to significantly reduce the growth of murine sarcoma S180 and hepatocarcinoma H22 subcutaneous allografts after daily intragastric administrations for ten days at the doses of 40, 60, or 80 mg/Kg. The highest percentage of inhibition (more than 40%) was achieved with 80 mg/Kg of the compound [68]. Later, Martinez-Diez et al. reported in vivo anticancer activity of the polyketide PM060184 (or Plocabulin) isolated from the sponge *Lithoplocamia lithistoides* [69]. This compound belongs to the class of tubulin-binding agents and is able to bind to a different binding site of β-tubulin, rather than the commonly known binding site vinca domain [101]. PM060184 drastically reduced tumor volumes in MDA-MB-231 (breast), HCT-116 (colon), H-460 (NSCLC), HGC-27 (gastric), 22RV1 (prostate), and Caki-1 (renal) subcutaneous xenografts when mice were treated i.v. once per week for three weeks with 16 mg/Kg of PM060184 [69]. Interestingly, PM060184 has gone through clinical trials and is under evaluation in an ongoing phase II clinical trial in advanced colorectal cancer patients (NCT03427268) and in a phase I clinical trial in combination with gemcitabine in patients with multiple solid tumors (NCT02533674).

Other promising chemicals derived from sponges with potential use in humans are the compounds Dictyoceratin-A and -C isolated from the Indonesian marine sponge *Dactylospongia elegans.* These two sesquiterpene phenols inhibited the growth of murine sarcoma 180 tumors by 90% after being orally administrated at the dose of 50 mg/Kg every two days for two weeks [70]. Additionally, the sphingolipid-like semi-synthetic compound Rizochalinin, a derivative from Rhizochalin, a bioactive chemical isolated from the marine sponge *Rhizochalina incrustata,* apart from inducing potent cytotoxicity with an IC_50_ in the low micromolar range in human prostate cancer cells, also impaired in vivo tumor growth of human prostate PC-3 and 22Rv1 xenografts by 27% and 46.8% respectively, after being i.p. administered at 1.8 mg/Kg/day without apparent toxicity [71]. Later, Huang et al. demonstrated anticancer activity of a novel compound, 6-Chloro-2-methoxy-N-(phenylmethyl)-9-acridinamine (BA), isolated from a marine sponge (species not described). BA inhibited in vitro cell proliferation and in vivo tumor proliferation in human liver carcinoma SMMC-7221 cells and SMMC-7221 subcutaneous xenografts. In vivo anticancer activity was assessed in mice administered i.p. with 5 or 10 mg/Kg of BA once a day for five consecutive days [72]. Notably, 10 mg/Kg of BA was more efficient than 10 mg/Kg of 5-fluorouracil at reducing tumor growth [72]. Recently, our research group identified two marine sponge compounds isolated from Western Australian sponges: Crambescidin 800 and Aurantoside C from the species *Monachora viridis* and *Manihinea lynbeazleyae*, respectively [73,74]. Interestingly, both compounds presented higher selectivity for triple negative breast cancer (TNBC) cells compared to normal cells with IC_50s_ in the low micromolar range and induced cell cycle arrest and apoptosis. Moreover, both Crambescidin 800 and Aurantoside C were at least ten times more effective at inducing cytotoxicity than the clinically approved drugs for TNBC, cisplatin, and doxorubicin [73,74]. Aurantoside, especially, showed a 35-fold change in potency compared to cisplatin [74].

### 3.2. Tunicates

Tunicates belong to the subphylum *Tunicata*, and its name derives from “tunic”, the outer covering of these animals that acts as an exoskeleton. Various species of these subphylums are commonly known as ascidians, sea squirts, sea porks, sea livers, or sea tulips. Most adult tunicates are sessile and permanently attached to rocks or fixed on the ocean floor. Thus, they rely on their natural defense against predators using an arsenal of toxins, ranging from cyclic peptides to aromatic alkaloids, inducing various biofunctional properties [75]. Many of these compounds are secondary metabolites produced directly or supported by symbiont bacteria, which ensures the defense and survival of their tunicates hosts [102].

Cyclic depsipeptides are a type of compound commonly found in ascidians, synthesized by ribosome independent mechanisms, and they are generally produced by symbiont microbes. The *Didemnidae* family of tunicates are the higher producers of these types of bioactives. Didemnin B (Figure 2), which belongs to the *Didemnidae* family, possesses the most potent biological activity. It was first isolated from the ascidian *Trididemnum solidum* in 1981 and has strong antiviral and anti-proliferative properties [81] as well as potent immunosuppressant activity [103]. It was able to block protein synthesis by direct binding to eukaryotic elongation factor 1A (eEF1A) [104]. Also, Didemnin B was found to induce apoptosis in proliferating lymphocytes and a panel of permanently transformed cell lines [105]. It has also showed potential benefit in a patient with pancreatic adenocarcinoma in a phase I clinical trial [106]. This evidence supported the evaluation of Didemnin B in phase II clinical trials in the U.S., being the first marine product to be approved. However, after trial completion against several cancer types (kidney, ovarian, and breast cancer), many severe side effects were observed, such as anaphylactic reactions and neuromuscular toxicity. For this reason, posterior human trials involving Didemnin B were discontinued [107]. Despite the initial drawback of Didemnin B, strategies focused on the search of other potent analogs were substantially increased. This is the case for Dehydrodidemnine B, commonly known as Aplidine or Plitidepsin (marketed as Aplidin^®^ by the company PharmaMar SA, Spain). This marine compound was initially isolated from the ascidian *Aplidium albicans* and shown to be the most powerful bioactive compound of the genus. The depsipeptide Dehydrodidemnine B exerted its antiproliferative activity similar to Didemnin B but with higher potency, showing an IC_50_ lower than 10^−8^ M on primary cultured murine Ehrlich mammary carcinoma cells [76]. Also, it reduced in vivo tumor growth when 2.5 mg were injected daily i.p. Currently, Dehydrodidemmine B is a promising marine compound used in many clinical trials (see Section 4). Another group of interesting depsipeptides are Tamandarins. They also belong to the *Didemninae* family of ascidians found in Brazil waters. Tamandarins showed potent cytotoxic activity in the human pancreatic cancer cells BX-PC3, prostate cancer cells DU-145, and head and neck carcinoma cells UMSCC10b [77]. When synthetically made, Tamandarins (A and B analogs), exerted the highest inhibitory growth potency (IG_50_) observed in this type of compounds. The IG_50s_ were between 1 to 4 nM, while Didemnin B was 13 nM. [107]. Other interesting cyclic compounds are the bicyclic peptide Vitilevuamide from *Didemnum cuculiferum* and the peptide Diazonamide from *Diazona angulata*. Vitilevuamide was found to be cytotoxic in several tumor cell lines (IC_50_: 6–311 nM) and to be a potent inhibitor of tubulin polymerization at 5.6 µM in a cell-based screen [78]. Diazonamide, also named DZ-2384, exhibited potent antitumor activity in models of multiple cancer types and lacked neurotoxicity in rats. Diazonamide binds the vinca domain of tubulin, causing the straightening of curved protofilaments [79].

Alkaloids belong to another big group of bioactive compounds isolated from ascidians with promising anticancer properties. One example is Trabectedin (Ecteinascidin-743 or ET-743), initially isolated from *Ecteinascidia turbinate*, and described to elicit impressive in vivo antitumor activity [80]. Trabectedin was rapidly obtained through semi-synthetic approaches [108] and was the first drug introduced to the market, commercialized with the name of Yondelis^®^ (PharmaMar SA). Trabectedin is currently under intense clinical investigation (see Section 4). During synthesis, other compounds with similar activity were created, such as Phthalasdicin and Lurbinectedin [109]. Other interesting subfamilies of alkaloids are Cystodytins, Styelsamines, Diplamines, and Ascididemins. Ascididemins, as well as Meridine, have been described to inhibit telomerase activity. A large subfamily of amino acid-derived alkaloids includes Euristomins and Lamellarins. Eudistomins are tryptophan-derived alkaloids with potent cytotoxic activity. They were originally isolated by Rinehart’s group [81] from the Caribbean tunicate *Eudistoma olivaceum*. Eudistomin C is found to exert antitumoral and antiviral activity, targeting the 40S ribosome subunit and inhibiting protein translation [110]. Eudistomin inhibited in vivo tumor growth of murine leukemia P338 allografts when injected i.p. at doses ranging from 0.03 to 8 mg/Kg [81]. Lamellarins are derived from the amino acids phenilalanine or tyrosine and are found in the mollusk *Lamellaria sp.* as well as in the Didemnid ascidian and sponges. They possess cytotoxic activity, with IC_50_ values ranging from nanomolar to micromolar [82]. Among them, lamellarin D is the most potent inhibitor of both nuclear and mitochondrial topoisomerase I, but is also capable of directly interfering with mitochondria function to trigger cancer cell death [82,111]. Lamerallins have also been proven to affect other cancer targets such as protein kinases and drug efflux pumps. Indole-based alkaloids such as Staurosporine and the synthetic analog 7-hydroxystaurosporine (UCN-01) have been reported to inhibit several kinases involved in cancer survival signaling pathways and cell cycle. In particular, UCN-01 induces strong inhibition of the phosphokinases AKT, PKC, CDK, and the checkpoint kinase Chk1 at nanomolar doses [112]. Bistramide A (bisA or bistratene A) is a cyclic polyether which was first isolated from *Lissoclinum bistratum*. Its antiproliferative effects can be explained by the direct binding to monomeric G-actin in a 1:1 ratio (K_d_ = 7 nM). This resulted in the inhibition of actin polymerization and consequent disruption of the actin cytoskeleton [113].

Polyketides are a large group of marine compounds where the most known members are Mandelalides. Mandelalides A–D were isolated from new species of the ascidian *Lissoclinum* collected from South Africa [114]. The glycosylated forms of Mandelalides B and E were found to be cytotoxic to proliferating NCI-H460 lung cancer, HeLa, U87-MG glioblastoma, and HCT116 colon cells [85]. Their cytotoxic activity is mediated by the inhibition of aerobic respiration. The same effect has been observed in vitro in isolated mitochondrias. Also, the sustained inhibition of mitochondrial function by low concentrations of mandelalides (1–300 nM) was sufficient to trigger cell death in HeLa cells through a caspase-dependent mechanism [115].

### 3.3. Mollusks

Mollusks, also spelled molluscs, are invertebrates possessing a soft body totally or partially covered by a calcium carbonate shell. The phylum *Mollusca*, which comprises around 85,000 species, is grouped into two subphyla: the Auculifera, which is further subdivided into two classes (Aplacophora and Polypalcophora), and the Conchifera, which is further grouped into five classes (Monoplacophora, Gastropoda, Cephalopoda, Bivalvia, and the Scaphopoda) [116]. A number of mollusks metabolites, with a majority of them classified under Gastropoda, have been identified to surmount cancer cell resistance to chemotherapy.

Jorumycin (Figure 2), a highly potent and complex pentacyclic compound, was isolated from the exterior layer and mucus of the shell-less marine gastropod mollusk *Jorunna funebris*. Initial studies carried out by Fontana et al. established antitumor activity with complete inhibition of cell survival at 50 ng/mL in mouse fibroblast cells and a higher potency in murine leukemia cells (P338), human lung alveolar cancer cells (A549), human colorectal cancer cells (HT29), and human melanoma cells (MEL28), with an IC_50_ of 12.5 ng/mL in all these cell lines [86]. A significantly higher level of potency was observed by Satio et al. using the semisynthetic version of Jorumycin, obtaining IC_50_ = 0.57, 0.76, and 0.49 nM in human colon cancer cells (HCT116), human lung cancer cells (QG56), and human prostate cancer cells (DU145), respectively [87]. In the process of analyzing potent analogs, Lane et al. confirmed inhibition with Jorumycin at a low nanomolar range, similar to previous studies [117]. Jorumycin possesses a similar chemical structure to Trabectedin isolated from tunicates, which is discussed in Section 3.2. The central pro iminium ion present in Jorumycin leads to covalent modifications causing cell death [118].

Dolastatins and, in particular, Dolastatin 10, originally isolated from the *Dolabella auricularia* and posteriorly from the cyanobacteria *Symploca sp.* [16], are very interesting cytotoxic compounds. *Dolabella auricularia* is a large sea slug classified as an opisthobranch gastropod mollusk. Dolastatin 10 displayed promising anti-proliferative properties at the time of isolation, with an ED_50_ of 4.6 × 10^−5^ ng/mL in the murine leukemia cells P338 and with less potency, but still promising, in the cancer cells OVCAR-3 (ovarian), SF-295 (glioma), A498 (kidney), NCI-H460 (lung), KM20L2 (colon), and SK-MEL-5 (melanoma) [119]. Dolastatin 10 was identified as having antimitotic activity as a result of the impediment of Guanosine triphosphate (GTP) hydrolysis, tubulin polymerization, and nucleotide exchange [120]. A study also linked the prevention of apoptotic function with Dolastatin 10 in small cell lung cancer [121]. Apart from the cancer cell lines described above, Dolastatin 10 also elicited in vitro antitumoral effects in the human colon adenocarcinoma cell line LoVo and the HeLa-derived cell line KB with IC_50_ of 0.052 and 0.076 nM, respectively [16]. Despite Dolastatin 10 being discontinued at phase II of clinical trials, several other analogs such as Dolastatin 15 and Auristatin were identified, with Dolastatin used as the parent compound [122]. With an IC_50_ ranging from 0.5 to 1 nM, Dolastatin 15 induced apoptosis resulting in repressed growth in human myeloma cells [89].

Another interesting compound originated in mollusks is Kahalalide F. Kahalalide F was originally isolated from *Elysia rufescens* by Hamann and group [123]. This cyclic depsipeptide has been established to possess high in vitro cytotoxic activity against human breast cancer cells H5578T (0.162 µM) and Hs-578T (IC_50_ = 0.479 µM), human non-small cell lung adenocarcinoma cells A549 (IC_50_ = 0.135 µM) and human colon cancer cells (IC_50_ = 0.162−0.288 µM) [90]. Through the inhibition of the PI3K-AKT pathway, the cytotoxic activity of Kahalalide F (KF) resulted in cell death in cancer cells [124]. Also, KF potently reduced in vivo tumor growth and navigated through phase I and II clinical trials for advanced solid cancers [125,126], albeit with limited activity. Despite being discontinued from clinical trials, it paved the way for other compounds such as the deptispeptide Elisidepsin which was derived from KF synthetically. Currently being evaluated in phase II of clinical trials, Elisidepsin displayed cytotoxic activity ranging from 0.4 to 8.8 µM in a panel of 23 cancer cells, including human breast, colon, head and neck, liver, lung, melanoma, ovarian, pancreas, and prostate cancer cells [91]. Elisidepsin was also evaluated with other chemotherapies including lapatinib, 5-Fluorouracil, and oxaliplatin, resulting in a synergistic effect [91]. Another cyclic desipeptide compound, Kulokekahilide-2, was isolated from the cephalaspidean mollusk *Philinopsis speciosa*. The potency and selectivity of this antitumor compound were investigated over several cancer cells, including murine leukemia P388 cells, human ovarian cancer SK-OV-3 cells, and human melanoma MDA-MB-435 cells, with IC_50_ ranging from 4.2 to 14.6 nM [92]. In contrast, Kulokekahilide-2 showed an IC_50_ of 59.1 nM in the non-cancerous rat cell line A-10, suggesting its low cytotoxicity in normal cells.

Along with the above mentioned mollusk-derived compounds, there are other compounds such as Depatuxizumab mafodotin (ABT-414), Enfortumab vedotin (AG-22ME), and Polatuzumab vedotin (DCDS-4501A) which are either currently being investigated in phase III clinical trials or approved by the FDA. These compounds are antibody drug conjugates which consist of a mollusk-derived drug with an antibody specific for cancer cells. This relatively novel class of compounds is further discussed in Section 4.

### 3.4. Bryozoans

Bryozoans are small aquatic invertebrates living in colonies. The colonies usually have a skeleton of calcium carbonate. Despite accounting for approximately 5000 species, Bryozoans are a relatively unknown group of organisms. The phylum *Bryozoa* is classified into three main classes: Stenolaemata, Phylactolaemata, and Gymolaemata. The class Gymolaemata is further subdivided into the orders Cheilostomatida and Ctenostomatida [127]. Due to heavily calcified bryozoan samples and other limitations (discussed in Section 5), isolation of secondary metabolites from Bryozoans has been indicated to be taxing, which is one of the reasons for comparatively less studies in this phylum.

However, several bryozoan-derived compounds have been identified to possess antitumoral properties. This is the case for Tambjamine. Tambjamine is a class of cytotoxic alkaloid originally isolated from *Virididentula dentate*, formerly known as *Bugula dentata* (first discoverer: Lamoroux (1816)), by Carbone and group [128]. Aldrich et al. later synthesized Tambjamine K, a derivative of Tambjamine A, creating a more potent antitumor compound [93]. Unnatural analogs of Tambjamine K, namely, compounds *12*, *13* and *14*, displayed anti proliferative activity against breast cancer MDA-MB-231 cells and colon cancer HCT116 cells with IC_50_ ranging from 0.36 to 15.3 µM [93]. Recently indole-based analogs of Tambjamine were synthesized and were investigated in vivo in small-cell lung cancer DMS53 xenografts. Compounds *1* and *2* displayed IC_50_ values below 10 µM [94]. It showed that apoptosis was the primary mechanism that resulted in cell death. Moreover, compounds *1* and *2* reduced tumor burden in mice bearing subcutaneous and orthotopic DMS53 xenografts when injected i.p. at 6 mg/Kg, with compound *2* being the most potent with no obvious toxicity observed [94].

Bryostatins are the most promising anticancer lactone compounds. They were originally isolated from the Cheilostome bryozoan *Bugula neritina* by Pettit et al. [129]. Through the regulation of protein kinase C, the macrocyclic lactone Bryostatin 1 (Figure 2) has been identified to play a vital role in tumor cell growth. Anti-tumor activity was identified (IC_50_ = 0.25 nM) in the murine leukemia cell line P338 [95]. Bryostatin 1 was under investigation in phase II clinical trials for the treatment of patients with metastatic colorectal cancer [130]. However, no partial or complete responses were observed. Combinatorial studies with other agents such as cisplatin along with Bryostatin 1 have also been investigated in phase II clinical trials in recurrent or persistent carcinoma of the ovary with modest responses observed in some patients [131]. Despite being discontinued in the clinic, Bryostatin 1 has led to several other studies analyzing its possible synergistic effects with other agents. Moreover, several other analogs of Bryostatin 1, such as the macrocyclic lactones Bryostatin 5 and Bryostatin 8, have been studied and have demonstrated significant antitumoral activity in vivo against melanoma K1735-M2 allografts [96]. According to Kraft’s study, the three bryostatins showed similar inhibition of tumor growth when animals were treated i.p. with 1 µg/injection. However, animals administered with Bryostatin 5 and 8 had lower weight loss compared to Bryostatin 1 [96]. Bryostatin 5 has also displayed promising anti-tumor activity by inducing macrophage-like cell differentiation in human myeloid cells at a concentration of 10 nM [132]. Such an effect was potentiated by vitamin D3 [132].

## 4. Marine Compounds Approved for Cancer Treatment

The marine ecosystem is the source of a large number of drugs used for cancer treatment. Up to now, there are eight drugs approved by the regulatory institutions FDA, EMEA, and ATGA: Cytarabine (Cytostar-U^®^, Depocyt^®^), Fludarabine (Fludara^®^), Nelarabine (Arranon^®^), Trabectedin (Yondelis^®^), Eribulin mesylate (Halaven^®^), Brentuximab vedotin (Adcetris^®^), Plitidepsin (Aplidin^®^), and Polatuzumab vedotin (Polivy^®^) (Figure 1). Their commercial name, mechanism of action, active derivative, origin, target, and further information are summarized in Table 8. All of them are being further tested in tens of on-going clinical trials for a more diverse scenario of cancer types as the ones they were initially approved for.

The first experiments with Cytarabine took place in 1961, where this compound inhibited the growth of several mouse tumors [133]. In 1964, the first clinical trials began and finally, in 1969, after the conclusions of several oncologic groups, Cytarabine (Cytosar-U^®^) obtained FDA approval for the treatment of acute myeloid leukemia [10]. Rapidly, its clinical effectiveness became popular and its use was extended to the treatment of acute meningeal and lymphocytic leukemia, chronic myelogenous leukemia, and lymphoma [134]. The therapeutic relevance of Cytarabine is reflected by its inclusion in the Model List of Essential Medicines of the World Health Organization (https://www.who.int/medicines/publications/essentialmedicines/en/). Chemically, Cytarabine is named 1β-arabinofuranosylcytosine and is an antimetabolic agent that interferes with DNA synthesis. Cytarabine is an analog of 2’-deoxycitidine that inhibits DNA polymerization after being incorporated into the DNA as a fraudulent deoxynucleotide.

Trabectedin (Yondelis^®^) is another institutionally approved marine drug, initially approved by the EMEA in 2007. Its use was investigated for patients with liposarcoma and leiomyosarcoma who failed to be administered with doxorubicin or ifosfamide or were unsuited to receive these agents. Despite Trabectedin lacking consistent results, the Committee for Medicinal Products for Human Use concluded that Trabectedin’s benefits were greater than its risks and it was recommended for marketing under “exceptional circumstances”. Finally, in 2015, the FDA approved Trabectedin based on the excellent results of a phase III study comparing Trabectedin with dacarbazine in terms of efficacy for the same soft-tissues sarcomas [135]. Moreover, Trabectedin showed significant benefits used in combination with pegylated liposomal doxorubicin in a phase III study (OVA-301) in patients with recurrent ovarian cancer [136]. As a result, EMA approved Trabectedin for its use in this type of cancer in 2009. Now, Trabectedin is in phase III for peritoneal, fallopian tube cancer (NCT01846611), and for other soft sarcomas (NCT02672527). Trabectedin presents a complex mechanism of action affecting both tumor cells and the tumor microenvironment. In particular, Trabectedin inhibits oncogenic progression by blocking DNA polymerase II, covalently binding to amino groups in the minor groove of DNA that affects oncogenic transcription, and impairing DNA repair mechanisms. It also modulates the production of cytokines and chemokines in tumor-associated macrophages [137].

Eribulin mesylate (Halaven^®^) is a fully synthetic macrocyclic ketone analog of the marine natural product Halichondrin B. Eribulin and other analogs showed sub-to-low nanomolar growth inhibitory potential. Eribulin and Halichondrin B were able to induce mitotic spindle disruption and mitotic blockade, both in vitro and in vivo in various human tumor models [62]. Preclinical studies in human breast cancer models showed different antitumoral-based properties for Eribulin such as tumor-associated vascular remodeling and tumor hypoxia mitigation, both leading to the reversal of tumor aggressiveness [138] and epithelial–mesenchymal transition and inhibition of metastasis [139]. In 2010, this mesylate salt was approved by the FDA to treat patients with metastatic breast cancer who had received at least two prior chemotherapy regimens for late-stage disease. Later, in 2016, FDA approved Halaven^®^ for the treatment of inoperable liposarcoma after the findings of a phase III trial, which showed increased overall survival in patients assigned to Eribulin compared with those treated with dacarbazine (13.5 versus 11.5 months) [140]. Now, Eribulin is also being evaluated in diverse phase III trials, including non-small cell lung cancer (NCT01454934), soft tissue sarcoma (NCT01327885), and HER2-negative breast cancer brain metastases (NCT03613181).

Brentuximab vedotin (Adcetris^®^) is an antibody drug conjugate (ADC) composed of a monoclonal antibody that targets the cell membrane protein CD30, linked with the antimitotic agent monomethyl auristatin E (MMAE). The antibody binds to CD30 on the surface of malignant cells, delivering MMAE in the tumor environment through a complex lysosome-mediated mechanism. This ensures the specificity and selectivity of the antitumoral activity of MMAE, a synthetic linear peptide belonging to the family of Dolastatins. Auristatins are antimitotic toxins derived from the natural product Dolastatin 10 found in the sea hare *Dolabella auricularia*. Dolastatins inhibit microtubule assembly by impairing tubulin formation, which leads to cell division disruption and apoptosis [141]. In 2011, FDA approved the use of Brentuximab vedotin for the treatment of both Hodgkin lymphoma (HL) and anaplastic large cell lymphoma (ALCL) after the good results of two phase II trials [142,143]. These results included objective cancer remission in 75% and tumor reduction in 94% of patients with refractory or relapsed HL and 86% of remission and 97% of tumor shrinkage in the patients with refractory of relapsed ALCL. Afterwards, in 2018, the FDA granted an expansion for the utilization of Brentuximab vedotin as the first-line treatment of stage III and IV in HL patients in combination with chemotherapy. Now, the study of Brentuximab vedotin has been extended to other types of lymphomas such as B-cell and T-cell lymphomas (NCT01421667) and solid tumors such as testicular cancer (NCT02689219).

Plitidepsin (Aplidin^®^), developed by PharmaMar, belongs to the chemical compound family of didemnins, the cyclic depsipeptides originally isolated from tunicates. Plitidepsin has been shown to induce cell-cycle arrest and apoptosis via activation of JNK and p38 mediated by oxidative stress [144]. Other studies hypothesize that the interaction of Plitidepsin with the transcription factor eEF1A2 is the primary mechanism of anticancer action. Such interaction inhibits the pro-oncogenic behavior of eEF1A2. Interestingly, eEF1A2 is overexpressed in many malignancies, including multiple myeloma (MM) [145]. In vitro studies showed that Plitidepsin elicits strong cytotoxic activity in several cancer cell lines, presenting IC_50_ values ≤1 nM [146], and in vivo studies demonstrated the potent antiproliferative effects of Plitidepsin in xenograft models of MM [147]. Nevertheless, Plitidepsin can possess limited antitumor activity as a single agent in several malignancies, such as non-cutaneous peripheral T-cell lymphoma, melanoma, and MM [148]. Despite its limitations, in 2018, the ATGA approved Plitidepsin for anticancer treatment in patients affected by relapsed/refractory MM, making it the first-in-class anticancer agent approved for treatment. Results from a phase III study known as ADMYRE [149] encouraged the use of Plitidepsin combined with dexamethasone. In this clinical trial, MM patients treated with Plitidespin + dexamethasone experienced an increase in overall survival compared to dexamethasone alone (11.6 months vs. 8.9 months). Importantly, the safety profile did not overlap with the toxicity observed with other agents used in MM. Currently, Plitidepsin is under clinical investigation for other hematological cancers such as angioimmunoblastic T-cell lymphoma (NCT03070964), leukemia (NCT00780143), and solid tumors such as prostate cancer (NCT00780975).

Polatuzumab vedotin (Polivy^®^) is another marine-derived drug recently approved by the FDA (June 2019) for the treatment of relapsed or refractory diffuse large B-cell lymphoma. Polatuzumab vedotin is an ADC that consists of the drug monomethyl auristatin (MMA) E and an antibody targeting CD79b, an exclusive marker of B cells. The drug approval was motivated by the results from the study GO29365 (NTC02257567), which showed a complete response rate of 40% versus 18% in patients treated with Polatuzumab vedotin + bendamustine + rituximab in comparison with those patients not treated with Polatuzumab vedotin.

Fludarabine is an antineoplastic agent used in the treatment of hematological malignancies, particularly chronic lymphocytic leukemia (CLL) and indolent B-cell lymphoma. It was approved in 2008 by the FDA for the treatment of relapsed CLL and it is now commercialized under the name Fludara^®^. Fludarabine is a drug precursor originally found in sponges. It is converted to the purine analog named F-ara-A. Its bioavailability is enhanced when it gets phosphorylated and inhibits cell proliferation through the inhibition of DNA polymerase and ribonuclease reductase [150].

Nelarabine (Arranon^®^) was approved in 2005 by the FDA for the treatment of patients with relapsed or refractory T-cell acute lymphoblastic leukemia and T-cell lymphoblastic lymphoma after at least two prior regimens [151]. It is a sponge compound, chemically characterized as an analog of arabinosylguanine nucleotide triphosphate (ara-GTP). Therefore, it is a purine nucleoside. Nelarabine inhibits DNA synthesis and consequently induces cytotoxicity.

A vast list of antibody-MMA derivatives, either E or F forms, has emerged as a successful strategy to deliver antimitotic drugs to more targeted environments. There are currently two ADC in phase III clinical trials. One of them is Depatuxizumab mafodotin (ABT-414), the monomethyl auristatin F targeted with an EGFR monoclonal antibody, being evaluated in glioblastoma multiforme presenting EGFR amplification (NCT02573324). The other ADC is Enfortumab vedotin (ASG-22ME), the monomethy auristatin E conjugated with a monoclonal Nectin-4 antibody which recognizes Nectin-4, a type I transmembrane protein whose gene is found in copy number gain in cancer, especially in breast and urothelial cancer [152]. Enfortumab vedotin is under evaluation in patients with locally advanced or metastatic urothelial cancer (NCT03474107).

Other drugs different from ADC have also demonstrated to be useful as anticancer treatment. One example is Plinabulin, formerly named NPI-2358. It was originally found in fungi and inhibits tubulin polymerization. It is now being investigated in the DUBLIN-3 phase III trial for non-small-cell lung cancer (NSCLC) (NCT02504489), with results yet to be reported. Lurbinectedin (PM01183), similar to Trabectedin, is a selective inhibitor of RNA polymerase II. Tumors that are highly dependent on transcription, such as sarcomas, triple negative breast cancers, and small cell lung carcinomas, are very sensitive to the drug. Lurbinectedin has shown promising results in a phase II trial (NCT02454972) [153] as a second-line treatment for small cell lung cancer. Currently, it is under evaluation in phase III trials for ovarian cancer (NCT02421588) and small cell lung cancer (NCT02566993). Finally, the bacterial compound Marizomib, also known as Salinosporamide A (NPI-0052), is a potent proteasome inhibitor that is being investigated in a phase III clinical trial (NCT03345095) in newly diagnosed glioblastoma patients. The previous first generation of proteasome inhibitors such as Bortezomib has shown promising results in a wide range of human malignancies including melanoma and endometrial carcinoma [154,155,156,157,158,159,160].

Finally, a broad range of marine-derived compounds are currently in phase I or II of clinical development, such as Tisotumab vedotin, Zalipsis, Glembatumumab, AGS-16C3F, Telisotuzumab vedotin, PM060184, PSMA-ADC, Lifastuzumab vedotin, Pinatuzumab vedotin, Indusatumab vedotin, Denintuzumab mafoditin, Belantamab mafoditin, Ladiratuzumab vedotin, RC48-ADC, CAB-ROR-ADC, CX2029, and Enapotamab vedotin. The list is very vast. Notably, some of them are being investigated for cancers with bad prognosis.

## 5. Limitations of Antitumoral Marine Compounds for their Clinical Development and Strategies to Overcome the Limitations

While the use of bioactives from marine organisms as the basis for the development of new promising drugs has opened a new era for scientists, there are several challenges that these compounds need to overcome to be able to successfully progress through the clinical development pipeline (Table 9). These major challenges can be grouped into the following categories: lack of sustainable supply, low production, poor technical infrastructure, structural complexity, correct taxonomic determination, moderate efficacy and high market value. For each of these obstacles, there are different strategies that are currently being undertaken or under investigation in order to overcome the challenges.

### 5.1. Lack of Sustainable Supply

Once a particular natural product has been isolated from a marine organism and is identified as the lead compound, the problem of sustainable supply is likely to occur. This is mainly due to the low production of the bioactive compounds of interest by the natural organism and/or difficulty faced during the isolation process. While a small amount of bioactive material is usually sufficient for an initial pharmacological evaluation, a much larger number of samples is needed for thorough characterization of the pharmacological activity. For instance, it is estimated that only 0.4% of the total active compound needed can be extracted from marine sponges, which is insufficient for the production needed in commercial quantities [161]. The manufacturing quantities required for preclinical development range from several grams to hundreds of grams and up to multi-kilograms quantities for clinical phases [162].

Another limiting factor for marine drug development is the unique and sometimes exclusive variability on the production of the organism itself. Recollection of bioactive compounds from the same organism may turn difficult since environmental conditions play an important part in their natural production. For example, the levels of bioactive metabolites excreted from marine sponges to protect themselves against environmental stress vary depending on many variables such as predator threat [163], microbes association [164], overgrowth of fouling organisms [165,166], or competition [167]. These unpredictable sea conditions make near impossible for the successful cultivation and maintenance of the isolated material under laboratory conditions.

The search for sustainable supply sources of bioactive compounds has led to the development of different alternatives for resupply such as the total synthesis or hemisynthesis of the original compound, the synthesis of synthetic analogs with manageable properties, or the design of a simplified synthesizable pharmacophore, which is the indispensable part responsible for drug activity [168,169,170]. The total synthesis of a bioactive compound ensures a large-scale supply of a rare bioactive compound and lead optimization through structure–activity relationship studies. However, many natural product scaffolds are complex and regarded as “privileged” scaffolds with multiple stereocenters that render their purification processes difficult to carry out [171,172]. On the other hand, hemisynthesis may be presented as a better solution for a bioactive compound’s resupply as it involves harvesting a biosynthetic intermediate from the natural source rather than the lead itself and converting it into the lead. For instance, Halichondrin B is a potent cytotoxic macrocyclic lactone polyether isolated from Japanese sponge *Halichondria okadai* in 1985 [173], representing a complex marine natural product with 32 stereocenters. Seven years later, Aicher and colleagues successfully produced a total synthesis of this compound [98]. Further structure–activity relationship studies highlighted a more potent and stable ketone analog, which is Eribulin, having 19 stereocenters. The synthesis of Eribulin mesylate encompasses a 62-step synthesis through reliable chemistry. This, combined with drug safety studies, led Eribulin to become the fourth marine-derived drug on the market approved by FDA for treating metastatic breast cancer [174,175].

However, it is worth noting that these chemical approaches used for the synthesis of marine chemicals are still at infancy, presenting themselves with their own challenges. Many biosynthetic compounds are non-specific, which may result in the production of multiple analogs [176]. In contrast, sometimes, short fragments or simplified structures of synthetic analogs may retain their biological activity or even improve their activity towards the target [168].

### 5.2. Low Production of Bioactive Compounds

The low natural production of a marine compound by the producer organism can seriously halter its clinical development. This low production is understandable given that a big proportion of the marine bioactives are synthetized intermittently as a result of environmental changes or predator attacks. Possible solutions to this low production are the manipulation of the metabolic conditions or the genetic engineering of marine organisms in the laboratory. For example, the change in the fermentation conditions of the actinobacteria *Salinospora tropica* undertaken by Potts et al. led to higher yields of Salinosporamide A production [177]. The authors achieved more than a 100-fold increase in bioactive production, reaching the yield of 450 mg/L by changing the formulation of the culture media [177,178].

The extraction process plays a crucial step during the isolation of biologically active constituents in marine organisms in order to achieve high-end yield and product quality. Many conventional methods of extraction have presented a number of drawbacks leading to lower yield and high-energy cost. A properly designed and implemented methodology of extraction such as enzyme-assisted extraction, supercritical-fluid extraction, microwave-assisted extracted, and pressurized-liquid extraction has been demonstrated to meet challenges and opportunities in algae producers [179].

### 5.3. Poor Technical Infrastructure

The deep exploration of the marine environment began around the 1970s when modern snorkeling and scuba diving were introduced, and later, the use of remotely operated vehicles in the 1990s until today [162,180]. Three common sampling approaches were encountered on the lookout for biologically active compounds: (i) unexploited sources of geographical or taxonomic groups; (ii) new taxa and/or regions of confirmed chemical diversity; or (iii) combination of both strategies [181]. The search for biologically active compounds was initially conducted in shallow coastal waters (<30 meters) by performing small selection of random marine organisms, as the deepest spots of the oceans (i.e., hydrothermal vents and sea mounts) were very difficult or almost impossible to reach even though they represent a community of highly promising organisms [2].

Traditional methods used to analyze marine biodiversity include morphological species identification and toxicological analyses, which are time-consuming, expensive, and possess low upscaling potential to resolve changes [182]. This makes the distinction and quantification of new species by morphologically comparing known close species a real challenge. The development of sophisticated technologies such as remote sensing to explore wide geographic areas or autonomous observation platforms for large temporal scales demonstrate higher advantages for data availability such as improved taxonomic resolution and the ability to retrieve real-time information.

However, these sampling facilities have an elevated cost that is unaffordable for most marine research laboratories, which makes such facilities impossible to be carried out, especially when the majority of biological diversity are found in underdeveloped countries [183]. Thus, international collaborations in this research field are necessary and highly encouraged to allow new discoveries to take place. At the same time, the use of this advanced technology still needs to be tested prior to its application for identifying new bioactivities in deep-sea habitats.

### 5.4. Structural Complexity of the Marine Compounds

The large structural variety of isolated marine bioactive compounds may contribute to the failure of synthetic production of such compounds where their complexity leads to the wrong assignment of chemical formula, planar connectivity, intramolecular bonds, or stereocenters [184]. This stirs up a supply problem towards the drug discovery process where a continuous supply of the particular lead compound is a necessity. Initial efforts have mainly been invested in harvesting metabolites from marine species which are easily accessible. However, these metabolites are available in such minor quantities that this presents a challenge for analytic or biological evaluations. For that, in order to better understand the natural scaffolding of these drug candidates, in silico screening programs have been carried out [185].

The development of refined analytical and spectroscopic methods such as nuclear magnetic resonance (NMR) and mass spectrometry (MS) has allowed de novo structure assignment that has led to the discovery of new chemical entities present in small concentrations [186]. Thanks to new technological advances, those techniques can now be performed in smaller amounts such as in 384- and 1534-well plates [187]. This has provided an improvement in the way information is obtained from natural products discovered in the deep ocean [188].

### 5.5. Correct Taxonomic Determination

Many marine species are difficult to access and cultivate under laboratory conditions. Also, sometimes the microorganisms that reside in the marine organism, such as in marine sponges and tunicates, are the source for producing the bioactive molecules and not the invertebrate marine host itself [189,190]. All this could attribute to an inaccurate identification of the marine species responsible for the marketable drug and its posterior drug development difficult. In the past, the growth of marine species in the laboratory and comprehensive morphological and physiological characterizations were strictly essential for the correct taxonomic determination of marine organisms. In contrast, today’s available genetic techniques, including sequencing and phylogenetic analysis, offer the solution for correct identification of marine microorganisms with high reliability and small amounts of marine extract needed. Amplification of 16S rRNA is frequently used for the identification and classification of marine prokaryotes. For example, Cristensen et al. successfully identified marine microorganisms from marine sediments and sponges living at different depths in certain areas of the coast of Florida by GRAM staining, 16S rRNA amplification, sequencing, and BLAST alignment [191]. The microorganisms were taxonomically classified as well. Amplification of 18S rRNA is usually used for the identification of phytoplankton and other microeukaryotes [192] and amplification of 23S rRNA for the identification of zooplankton species [193]. Another frequently used genetic approach is the amplification of mitochondrial DNA. These targeted genomic amplifications allow for a higher depth of genomic coverage and can be used for relatively low abundant organisms. Apart from amplification of a specific genomic region, scientists are currently using shotgun metagenomic methods across entire genomes to identify marine organisms [194]. This approach enables extensive knowledge of the microorganism’s metabolic function through the identification of large quantities of genes. The identification of secondary or specialized metabolites contributes to the correct taxonomic determination of microorganisms. This can be done by unique computational tools such as the “antibiotics and secondary metabolites analysis shell”, antiSMASH, which identifies biosynthetic gene clusters in microorganisms. Since its release in 2011, antiSMASH has widely been used and improved versions of it have been launched, such as antiSMASH version 5 [195].

### 5.6. Moderate Efficacy

The efficacy of marine-derived drugs resides on its ability to elicit profound antitumoral effects while reducing cytotoxicity in healthy tissues. The conjugation of potent marine drugs to specific antibodies recognizing antigens that are overexpressed in the cancer cell membrane and slightly expressed or absent in normal cells allows for selective and potent cell death in cancer cells. This strategy is followed by ADCs. These marine-derived ADCs have an improved anticancer activity compared to the respective “untargeted” marine drugs. One example is Brentuximab vedotin approved for cancer treatment (see Table 8 and Section 4). This ADC is the combination of a synthetic analog of Dolastatin 10, MMAE, and the monoclonal antibody anti-CD30. This ADC presented very high potency against CD30^+^ Hodgkin lymphoma and anaplastic large cell lymphoma cells in vitro, with IC_50_ of 3 to 50 pM, while non-expressing CD30 cells were ~1000 times more resistant [196]. In addition, the conjugate MMAE-CD30 showed higher potency, water solubility, and stability in physiologic conditions than its parent, Dolastatin 10 [196].

Nanoparticle encapsulation is another strategy that has been used to enhance half-life in circulation, solubility in aqueous media and tumor targeting, and to reduce the toxicity and immunogenicity of marine drugs, resulting in increased effectiveness against cancer. Nanoparticles have been successfully used to encapsulate a wide variety of chemotherapeutic drugs and to deliver tumor-targeting components [197,198,199,200]. Nanoparticles have also been deployed to co-deliver marine drugs with other chemotherapeutics to enhance antitumoral activity. For example, the combination of the marine drug Cytarabine and the anthracycline Daunorubicine in the molar ratio 5:1 induced a potent synergistic effect in vitro and in vivo in murine leukemia models [201]. For in vivo, the same combination ratio was encapsulated in liposomes, namely CPX-351, and showed superior therapeutic index and survival compared to free-drug cocktails [201]. This inspired the use of CPX-351 (VYXEOS^®^) in humans. A recent phase III clinical trial demonstrated a significant median overall survival and remission rate of CPX-351 compared to the conventional Cytarabine and Daunorubicine regimens in older patients with newly diagnosed secondary acute myeloid leukemia [202]. This study led to the FDA approval of CPX-351 in 2018. Also, Mirazchi et al. demonstrated more effective tumor growth reduction, tumor delivery, and lower toxicity of Fucoidan-made nanoparticles encapsulating the PI3K inhibitor BYL719 and targeted with P-selectin in the patient’s derived xenografts of NSCLC compared to free BYL719 [50].

Another powerful strategy that could enhance the efficacy of marine-derived drugs is the conjugation of marine compounds to cell-penetrating peptides and tumor homing specific peptides such as RGD peptides. Cell-penetrating peptides have the ability to improve the cellular penetrability of therapeutics through the plasmatic and/or nuclear membrane to reach the target, enhancing the efficacy of the marine drug. Similarly, the linkage of marine drugs to tumor homing peptides would allow a higher accumulation of the drug in the tumor site, enhancing its anticancer efficacy in a similar way as the antibody of ADCs does. Cell-penetrating peptides and RGD peptides have been extensively used to enhance the anticancer efficacy of therapeutic peptides and other agents [197,199,200,203,204]. However, their applicability in marine drugs has yet to be explored despite the fact that a large number of marine drugs are peptides, peptides derivatives, and small-size molecules. Interestingly, multiple marine organisms have been shown to expel membrane-active peptides (MAPs) with the intrinsic ability to disturb mammalian cell membranes [205], suggesting its promising utilization of these marine peptides as drug delivery systems.

Undoubtedly, investigations focused on the natural properties of marine chemicals and the conjugation of marine drugs to cell-penetrating and tumor homing peptides in a way that increases drug potency and selectivity are highly warranted.

### 5.7. High Market Value

One of the most commonly overlooked limitations during the synthesis and development of new natural products is the high market value. These have been well discussed by Martin and colleagues [162] who have highlighted the challenges faced during early development phases. Among some of the points that need to be taken into account to decrease the market value are the potential industrial use of the product, the final cost per kg of the final bioactive material, the desired formulation and preferred route of administration of the compound, the sustainability of the supply and manufacturing process, and how the product will reach the market chain. Thus, in order to overcome this market limitation, rigorous planning is anticipated in each step of development.

## 6. Concluding Remarks

The marine ecosystem is an invaluable source of anticancer compounds. Intriguingly, there is a higher percentage of bioactives exhibiting antitumoral properties of a marine origin compared to that of terrestrial origin. This is due to the often-immobile nature of marine organisms and the harsh and highly changing marine environment, which equips these organisms with a varied chemical repertoire to face predators, lack of nutrients, and new metabolic needs, with unique biological and cytotoxic properties. The phylum *Porifera* is especially prolific in the generation of anticancer drugs and in these organisms was discovered the first anticancer compound. Further synthesis approaches led to the production of Cytarabine, which was granted approval by the FDA in 1969 for the treatment of acute myeloid leukemia. After Cytarabine, seven other marine-derived drugs have been approved for cancer treatment; the past and current decades being the most productive at implementing marine-derived drugs in the clinic. Also, antibody-drug conjugates occupy an important space in the clinical evaluation of marine-derived anticancer drugs, given that their tumor-targeting components are able to improve tumor selectivity and drug potency while lowering damage in non-cancerous tissues. However, normal clinical development of marine-derived drugs could be threatened by several limitations including lack of sustainable supply, low production of bioactive compounds, structural complexity, and moderate activity. Such limitations have been identified and new strategies to overcome the limitations have been proposed, such as the full synthetic manufacture of marine drugs, the change in the cultivation conditions of the marine organisms in the laboratory, the use of new MS techniques, the encapsulation of drugs in nanoparticles, and the conjugation of drugs with functional peptides. Certainly, adopting these solutions would accelerate the clinical translation of promising anticancer compounds originating from the marine ecosystem, which holds an enormous and incalculable pharmaceutical value.

## Figures and Tables

**Table 1:** List of promising anticancer marine products from bacteria, actinobacteria, and cyanobacteria studied in pre-clinical studies and reviewed in this work.

Compound Name	Marine Organism	Species Name	Active Derivative	Cancer Model	In Vitro	In Vivo	IC50 In Vitro	Route of Administration In Vivo	Dose Used In Vivo	Ref.
Pyrroloformamide	Actinobacteria	*Streptomyces sp.*	Pyrrolinonodithiol	Human prostate cancer cell line PC-3M	✓	✗	1.67 nM	N/A	N/A	[20]
Cromomycin A2	Actinobacteria	*Streptomyces sp.*	Aureolic acid	Human melanoma cell line MALME-3M	✓	✗	16.7 nM	N/A	N/A	[21]
Anthracyclinone 4	Bacteria	*Micromonospora sp.*	Anthracyclinone	Human colon adenocarcinoma cell line HCT-8	✓	✗	6.2 µM	N/A	N/A	[22]
Coibamide-A	Cyanobacteria	*Leptolyngbya sp.*	Cyclic depsipeptide	Human lung cancer cell line NCI-H460 and mouse neuro-2a cells	✓	✗	< 23 nM	N/A	N/A	[23]
Lucentamycins A	Bacteria	*Nocardiopsis lucentensis*	Non-ribosomal peptides	Human colon carcinoma cell line HCT-116	✓	✗	0.20 µM	N/A	N/A	[25]
Mixirins A, B & C	Bacteria	*Bacillus sp.*	Non-ribosomal peptides	Human colon carcinoma cell line HCT-116	✓	✗	A: 0.68, B: 1.6, and C: 1.3 mg/mL	N/A	N/A	[26]
Ohmyungsamycins A&B	Actinobacteria	*Streptomyces sp.*	Non-ribosomal peptides	Human colon carcinoma cell line HCT-116	✓	✗	A: 0.359 µM, B: 12.4 µM	N/A	N/A	[27]
				Human lung cancer cell line A549	✓	✗	A: 0.551 µM, B: 15.6 µM	N/A	N/A	
				Human stomach cancer cell line SNU-638	✓	✗	A: 0.532 µM, B: 13.5 µM	N/A	N/A	
				Human triple negative breast cancer cell line MDA-MB-231	✓	✗	A: 0.688 µM, B: 12.7 µM	N/A	N/A	
				Human hepatic adenocarcinoma cancer cell line SK-HEP-1	✓	✗	A: 0.816 µM, B: 16.8 µM	N/A	N/A	
Urukthapelstatin A	Actinobacteria	*Mechercharimyces asporophorigenens*	Non ribosomal peptides	Human lung cancer lines A549, DMS114, and NCIH460	✓	✗	A519: 12 nM	N/A	N/A	[28]
				Human ovarian cancer cell lines OVCAR-3, OVCAR-4, OVCAR-5, OVCAR-8, and SK-OV3	✓	✗	0.828–0.846 nM for the rest	N/A	N/A	
				Human breast cancer cell line MCF-7	✓	✗		N/A	N/A	
Symplostatin 1	Cyanobacteria	*Symploca hydnoides*	Linear pentapeptide	Human colon adenocarcinoma cell line LoVo	✓	✗	0.34-0.50 nM	N/A	N/A	[16]
				HeLa-derived cell line KB	✓	✗	0.15-0.20 nM	N/A	N/A	
				Early stage colon adenocarcinoma #38	✗	✓	N/A	i.v.	3 mg/Kg	
				Early stage mammary adenocarcinoma 16/C	✗	✓	N/A	i.v.	1.25 mg/Kg	
				Human breast cancer cell line MDA-MB-435	✓	✗	0.15 nM	N/A	N/A	[17]
				Human ovarian cancer cell line SK-OV-3	✓	✗	0.09 nM	N/A	N/A	
				Multidrug resistant human ovarian cancer cell line NCI/ADR	✓	✗	2.9 nM	N/A	N/A	
				Early stage colon adenocarcinoma #38	✗	✓	N/A	i.v.	3 mg/Kg	
				Early stage mammary adenocarcinoma 16/C	✗	✓	N/A	i.v.	0.5, 0.25 mg/Kg	
TZT-1027	Cyanobacteria	*Symploca sp.*	Synthetic tetrapeptide. Dolastatin 10 derivative	Murine leukemia P338, melanoma B16, colon cancer colon 26 and sarcoma M5076 allografts	✗	✓	N/A	i.p. and i.v.	0.125–3 mg/Kg	[18]
				Human lung cancer LX-1 and breast carcinoma MX-1 xenografts	✗	✓	N/A	i.v.	0.5–2 mg/Kg	

**Table 2:** List of promising anticancer marine products from fungus, microalgae, and macroalgae studied in pre-clinical studies and reviewed in this work.

Compound Name	Marine Organism	Species Name	Active Derivative	Cancer Model	In Vitro	In Vivo	IC50 in Vitro	Route of Administration In Vivo	Dose Used In Vivo	Ref.
Brocazine G	Fungus	*Penicilliu m brocae MA-231*	Disulfide- bridged diketopip erazines	Human ovarian cancer cell lines A2780 and A2780-cisplatin resistant	✓	✗	A2780: 664 nM A2780 (CisR): 664 nM	N/A	N/A	[32]
Compound 2	Fungus	*Penicilliu m sp. FJ-1*	Sesquiterp enoid	Human osteosarcoma cell line MG- 63 and MG-63 xenografts	✓	✓	55 nM	i.g.	10 and 30 mg/Kg	[33]
Astaxanthin	Microalgae	*Haematoc occus pluvialis*	Keto- carotenoid	Induced colonic pre- neoplastic progression in rats induced by 1,2 dimethylhydrazine	✗	✓	N/A	Orally	15 mg/Kg	[39]
Chlorella sorokiniana extracts	Microalgae	*Chlorella sorokinia na*	Not specified	Human lung adenocarcinoma cell lines A549 and CL1-5 xenograft	✓	✓	A549: >40% cell death: 50 ng/mL CL1-5: >70% cell death: 250 ng/mL	Orally	50 mg/Kg	[40]
H3-a1	Macroalgae	*Hydroclat hrus clathratus*	Sulfated polysacch aride	Human acute promyelocytic leukemia cell line HL-60, human breast carcinoma cell line MCF-7. and human hepatocarcinoma cancer cell lines	✓	✓	Not foun d	i.p.	20 and 50 mg/kg	[41]
				Murine sarcoma S180 allograft	✓	✓	Not found	i.p.	20 and 50 mg/kg
DAEB	Macroalgae	*Enteromorpha intestinalis*	Sulfated polysaccharide	Murine sarcoma S180 cells and S180 allograft	✓	✓	5.6% cell death: 800 µg/mL	i.g.	100, 200, and 400 mg/kg	[43]
Grateloupia longifolia poly- saccharide (GLP)	Macroalgae	*Grateloupia longifolia*	Sulfated polysacchar ide	Human microvascular endothelial cell line	✓	✗	0.86 mg/mL	N/A	N/A	[44]
				HMEC-1						
				Human umbilical vein endothelial cell line HUVEC	✓	✗	0.64 mg/mL	N/A	N/A	
				Murine fibroblast cell line NIH-3T3	✓	✗	1.01 mg/mL	N/A	N/A	
				Human breast cancer cell line MDA-MB-435	✓	✗	1.77 mg/mL	N/A	N/A	
				Human gastric cancer cell line MKN-28	✓	✗	1.66 mg/mL	N/A	N/A	
				Human colon cancer cell line HCT-116	✓	✗	1.42 mg/mL	N/A	N/A	
				Human ovarian cancer cell line SK-OV-3	✓	✗	2.65 mg/mL	N/A	N/A	
				Murine sarcoma cell line S180 and S180 allograft	✓	✓	1.72 mg/mL	i.v.	200 mg/Kg	
Eucheuma serra aggluttinin	Macroalgae	*Eucheuma serra*	Sulfated polysaccharide	Murine colon cancer cell line Colon26 and Colon26 allograft	✓	✓	>10 µg/mL	i.v.	400 µg	[45]
SargA	Macroalgae	*Sargassum stenophyllum*	Sulfated polysaccharide	Murine melanoma cell line B16F10 and B16F10 allograft	✓	✓	<200 µg/well	s.c.	1.5 or 150 µg	[46]
Marine-derived sulfated poly- saccharide (MSP)	Macroalgae	Not specified	Sulfated polysaccharide	Human breast carcinoma cell line MDA-MB-231 and murine Lewis lung carcinoma	✓	✓	>200 µg/mL	i.p.	10 –80 mg/Kg	[47]
Ca-SP	Macroalgae	*Spirulina platensis*	Sulfated polysaccharide	Murine melanoma lung metastasis model B16- BL6	✗	✓	N/A	i.v.	100 µg	[48]
Fucoidan	Macroalgae	Not specified	Sulfated polysaccharide	Human breast carcinoma cell lines 4T1 and MDA- MB-231, and 4T1 xenograft	✓	✓	>120 µ/mL	Not specified	0.25 mg	[51]
				Human head and neck carcinoma cell line Cal- 33, Cal-33 xenograft and PDX H22	✗	✓	N/A	i.v.	7, 25, and 50 mg/Kg	[50]
				Murine Lewis lung carcinoma cell line LLC1 and LLC1 allograft	✓	✓	<6.25 µg/mL	Orally	15 mg/kg	[51]

**Table 3:** List of promising anticancer marine products from higher plants studied in pre-clinical studies and reviewed in this work.

Compound Name	Marine Organism	Species Name	Active Derivative	Cancer Model	In Vitro	In Vivo	IC50 in Vitro	Route of Administration in Vivo	Dose Used in Vivo	Ref.
ALE	Higher plant	*Acanthus ilicifolius Linn.*	Not specified	Murine Ehrlich ascites carcinoma	✗	✓	N/A	i.p.	2.5 mg/Kg	[56]
TCE	Higher plant	*Terminalia catappa L.*	Not specified	Human lung adenocarcinoma cell line A549	✓	✗	>100 µg/mL	N/A	N/A	[57]
				Murine Lewis lung carcinoma cell line LLC and LLC allograft	✓	✓	14.5 µg/mL	Orally	3 g/Kg	
Tagalsin C	Higher plant	*Ceriops tagal*	Dolabrane-type of diterpene	Human T cell leukemia cell lines Jurkat, SupT1, and Molt-4	✓	✗	<2.5 µM	N/A	N/A	[58]
				Human myeloma cell lines U-266 and PRMI-8266	✓	✗	<2.5 µM	N/A	N/A	
				Human lymphoma cell lines L1236 and KM-H2	✓	✗	<2.5 µM	N/A	N/A	
				T cells from acute myeloid leukemia patients	✓	✗	>0.5 µM	N/A	N/A	
				Human T cell leukemia line CEM and CEM xenograft	✓	✓	< 0.5 µM	i.p.	50 mg/Kg	
3-chlorodeoxylapachol	Higher plant	*Avicennia germinans*	Naphthoquinone	Human colon cancer cell line Col2	✓	✗	3.7 µg/mL	N/A	N/A	[59]
				Human prostate cancer cell line LNCaP	✓	✗	4.1 µg/mL	N/A	N/A	
				Human lung cancer cell line Lu1	✓	✗	8.3 µg/mL	N/A	N/A	
				Human telomerase reverse transcriptase- retinal pigment epithelium hTERT-RPE1	✓	✗	5 µg/mL	N/A	N/A	
				Human oralepidermoid carcinoma cell line KB and KB xenograft	✓	✓	3.2 µg/mL	i.p.	5 mg/Kg	
R. apiculata extract	Higher plant	*Rhyzophora apiculata*	Not specified	Murine melanoma mice model B16F10	✗	✓	N/A	i.p.	10 mg/Kg	[60]

**Table 4:** List of promising anticancer marine products from sponges studied in pre-clinical studies and reviewed in this work.

Compound Name	Marine Organis	Species Name	Active Derivative	Cancer Model	In Vitro	In Vivo	IC50 in Vitro	Route of Administration In Vivo	Dose used In Vivo	Ref.
Halichondrin B	Sponge	*Halichondria okadai*	Polyether macrolide	Murine melanoma cell line B16 and B16 allograft	✓	✓	0.093 ng/m L	i.p. and i.v.	2.5–20 µg/Kg	[61]
				Murine leukemia cell lines P338 and L-1210 and P338 and L-1210 allografts	✗	✓	N/A	i.p.	1.25–100 µg/Kg	
ER-076349 and ER-086526	Sponge	synthetically synthesized from Halichondrin B	Macrocyc lic ketone	Human promyelocytic leukemia cell line HL-60	✓	✗	0.41 nM	N/A	N/A	[62]
				Human histiocytic lymphoma cell line U937	✓	✗	0.22 nM	N/A	N/A	
				Human prostate cancer cell line LNCaP	✓	✗	0.25 nM	N/A	N/A	
ER-076349 and ER-086526	Sponge	synthetically synthesized from Halichondrin B	Macrocyclic ketone	Human prostate cancer cell line DU 145	✓	✗	0.70 nM	N/A	N/A	[62]
				Human colon cancer cell line DLD-1	✓	✗	0.75 nM	N/A	N/A	
				Human breast cancer cell line MDA-MB-435 and xenograft	✓	✓	0.14 nM	i.v.	0.25–1 mg/Kg	
				Human colon cancer cell line COLO205 and COLO205 xenograft	✓	✓	0.41 nM	i.p.	0.125–0.5 mg/Kg	
				Human melanoma cell line LOX and LOX xenograft	✓	✓	0.76 nM	i.p.	0.1–0.5 mg/Kg	
				Human ovarian cancer cell line NIH:OVCAR-3 and NIH:OVCAR-3 xenograft	✗	✓	N/A	i.v.	0.125–1 mg/Kg	
Girodazole	Sponge	*Pseudaxinyssa cantharella*	(1S,2S)-3-amino-1-(2-amino-1H-imidazol-5- yl)-2-chloropropan-1- ol;dihydrochloride	Murine leukemia cell line P388 and P338 and L1210 allografts	✓	✓	Not found	i.p.	Not found	[63]
				Murine mammary adenocarcinoma 16/C allograft	✗	✓	N/A	s.c.	Not found	
				Murine M5076 histiocytosarcoma	✗	✓	N/A	i.v.	Not found	
Agelasphin-11	Sponge	*Agelas mauritianus*	Galactosylceramide	Murine melanoma model B16	✗	✓	N/A	i.v.	0.1 mg/Kg	[64]
Pachymatismin	Sponge	*Pachymatisma johnstonii*	Glycoprotein	Human non-small cell lung cancer cell line N6 and N6 xenograft	✓	✓	Not found	Not found	Not mentioned	[65]
Naamidine	Sponge	*Fijian Leucetta*	2-aminoimidazole alkaloid	Human squamous cell carcinoma A431 xenograft	✗	✓	N/A	Not found	25 and 50 mg/Kg	[66]
Scalarane sesterterpne 1	Sponge	*Hyrtios erecta*	Sesterterpene	Murine lymphatic leukaemia cell line P338 and P338 allograft	✓	✓	14.5 ng/mL	i.p.	0.5–8 mg/Kg	[67]
				Human gastric cancer cell line MKN-1	✓	✗	57.7 ng/mL	N/A	N/A	
				Human gastric cancer cell lines MKN-7 and MKN-74	✓	✗	56 and 36.8 ng/mL	N/A	N/A	
B6. Derivative of Aldisin	Sponge	*Polymistia sp.*	Bromopyrrole	Human nasopharyngeal carcinoma cell line CNE	✓	✗	17.18 µg/mL	N/A	N/A	[68]
				Human breast carcinoma cell line MCF-7	✓	✗	11.30 µg/mL	N/A	N/A	
				Human hepatic carcinoma cell line HepG2	✓	✗	15.30 µg/mL	N/A	N/A	
				Human colon carcinoma cell line Lovo	✓	✗	3.83 µg/mL	N/A	N/A	
				Human hepatocarcinoma cell line BEL-7402	✓	✗	10.98 µg/mL	N/A	N/A	
				Human cervical epithelial carcinoma cell line HeLa	✓	✗	5.46 µg/mL	N/A	N/A	
				Murine sarcoma S180 and hepatocarcinoma H22 allografts	✗	✓	N/A	i.g.	40, 60, and 80 mg/Kg	
Plocabulin or PM060184	Sponge	*Lithoplocamia lithistoides*	Polyketide	Human ovarian cancer cell lines IGROV-1 and IGROV-1/ET	✓	✗	0.4 and 4 nM	N/A	N/A	[69]
				Human ovarian cancer cell lines A2780 and A2780/Dox	✓	✗	2.5 and 17 nM	N/A	N/A	
				Human colon carcinoma cell lines Lovo and Lovo/Dox	✓	✗	0.5 and 5 nM	N/A	N/A	
				Human breast carcinoma MDA-MB-231 xenograft	✗	✓	N/A	i.v.	16 mg/Kg	
				Human colon carcinoma HCT-116 xenograft	✗	✓	N/A	i.v.	16 mg/Kg	
				Human gastric cancer NGC-27 xenograft	✗	✓	N/A	i.v.	16 mg/Kg	
				Human non-small cell lung cancer H-460 xenograft	✗	✓	N/A	i.v.	16 mg/Kg	
				Human prostate cancer 22RV1 xenograft	✗	✓	N/A	i.v.	16 mg/Kg	
				Human renal cancer Caki-1 xenograft	✗	✓	N/A	i.v.	16 mg/Kg	
Dictyoceratin-A and -C	Sponge	*Dactylospongia elegans*	Sesquiterpene phenol	Murine sarcoma cell line S180	✗	✓	N/A	Orally	10–50 mg/Kg	[70]
Rhizochalinin	Sponge	*Rhizochalina incrustata*	Sphingolipid-like	Human prostate cancer cell lines DU145, LNCaP, and VCaP	✓	✗	DU145, LNCaP: <1.5µM, VCaP: <0.5 µM	N/A	N/A	[71]
				Human prostate cancer cell PC-3 and PC-3 and 22Rv1 xenografts	✓	✓	<1.5 µM	i.p.	1.8 mg/Kg	
BA	Sponge	Not specified	Acridinamine	Human liver carcinoma cell line SMMC-7221 and SMMC-7221 xenograft	✓	✓	<16 µM	i.p.	5 and 10 mg/Kg	[72]
Crambescidin 800	Sponge	*Monachora viridis*	Not specified	Human breast carcinoma cell line SUM149PT, SUM159PT, MDA-MB- 231, MCF-7, and ZR-75-1	✓	✗	6.02, 3.42, 5, 4.72, 8.09 µM, respectively	N/A	N/A	[73]
Aurantoside C	Sponge	*Manihinea lynbeazleyae*	Not specified	Human breast carcinoma cell line SUM149PT, SUM159PT, MDA-MB- 231, MCF-7, ZR-75-1, and T47D	✓	✗	0.81, 0.56, 0.61, 1.15, 1.91, 2.45 µM, respectively	N/A	N/A	[74]

**Table 5:** List of promising anticancer marine products from tunicates studied in pre-clinical studies and reviewed in this work.

Compound Name	Marine Organism	Species Name	Active Derivative	Cancer Model	In Vitro	In Vivo	IC50 In Vitro	Route of Administ Ration In Vivo	Dose Used In Vivo	Ref.
Didemmin B	Tunicate	*Trididemnum sp.*	Depsipeptide	Human permanent transformed cell lines HL-60, Daudi, Namalwa	✓	✗	1 µM: 94%, 3.4%, 19.2% apoptosis, respectively	N/A	N/A	[75]
				Human permanent	✓	✗	1 µM: 14.4%, 6.7%,	N/A	N/A	
				transformed cell lines BL-29, Naliaka, PDC-P1			7.9%			
				Human	✓	✗	1 µM: 18%, 3.3%, 90%,	N/A	N/A	
				Permanent transformed cell						
				lines Molt-4,			2%			
				Jurkat, MM96,			apoptosis,			
				and quiescent			respectively			
				PBMC						
Dehydrodidemnine B	Tunicate	*Aplidium albicans*	Depsipeptide	Murine Ehrlich ascitic mammary carcinoma cells and mouse model	✓	✓	<10 nM	i.p.	2.5 mg	[76]
Tamandarin A and B	Tunicate	Unidentified. Didemnidae family	Depsipeptide	Human pancreatic carcinoma cell line BX-PC3	✓	✗	<10 ng/mL	N/A	N/A	[77]
				Human prostate cancer cell line DU-145	✓	✗	<2.5 ng/mL	N/A	N/A	
				Human head and neck carcinoma cell line UMSCC10b	✓	✗	<5 ng/mL	N/A	N/A	
Vitilevuamide	Tunicate	*Didemnum cuculiferum*	Bicyclic peptide	Human colon cancer cell line HCT-116	✓	✗	6 nM	N/A	N/A	[78]
				Human lung adenocarcinoma cell line A549	✓	✗	124 nM	N/A	N/A	
				Human melanoma cell line SK-MEL-5	✓	✗	311 nM	N/A	N/A	
				Human kidney cancer cell line A498	✓	✗	311 nM	N/A	N/A	
				Chinese hamster ovary cells	✓	✗	3.1 µM	N/A	N/A	
				Murine leukemia P338 allograft	✗	✓	N/A	i.p.	6 –130 µg/Kg	
Diazonamide	Tunicate	*Diazona angulata*	Peptide	Human pancreatic cancer MIA PaCa-2, colon cancer HT-29, and MDA- MB-231-LM2 xenografts	✗	✓	N/A	i.v.	2.25–36 mg/m2	[79]
Trabectedin	Tunicate	*Ecteinascidia turbinata*	Alkaloid	In vivo cancer model. Not found	✗	✓	N/A	Not found	Not found	[80]
Eudistomin	Tunicate	*Eudistoma olivaceum*	Amino acid- derived alkaloid	Murine leukemia cell line L1210 and murine leukemia P388 allograft	✓	✓	0.015–0.26µg/mL	i.p.	0.03–8 mg/Kg	[81]
Lamellarin D	Tunicate	*Lamellaria sp.*	Amino acid- derived alkaloid	Human oralepidermoid carcinoma cell line KB	✓	✗	0.04 µM	N/A	N/A	[82]
				Human adenocarcinoma cell line A549	✓	✗	0.06 µM	N/A	N/A	
				Multidrug resistant human small cell lung cancer cell line H69AR derived from NCI-H69	✓	✗	0.4 µM	N/A	N/A	
				Human breast cancer cell line T47D	✓	✗	0.00008 µM	N/A	N/A	
				Triple negative breast cancer cell line MDA- MB-231	✓	✗	0.4 µM	N/A	N/A	
				Human liver cancer cell line HuCCA-1	✓	✗	0.08 µM	N/A	N/A	
				Human liver cancer cell line HepG2	✓	✗	0.02 µM	N/A	N/A	
				Human liver cancer cell line S102	✓	✗	3.2 µM	N/A	N/A	
				Human cervical epithelial carcinoma cell line HeLa	✓	✗	0.06 µM	N/A	N/A	
Staurosporine	Tunicate	*Eudistoma toealensis*.	Indolocarbazole alkaloid	Murine leukemia cell line P388	✓	✗	0.1 µM	N/A	N/A	[82]
				Human acute promyelocytic leukemia cell line HL-60	✓	✗	0.04 µM	N/A	N/A	
				Human leukemia cell line CMK	✓	✗	77% growth inhibition at 2 mg/mL	N/A	N/A	
				Human leukemia cell line HL-60	✓	✗	72% growth inhibition at 2 mg/mL	N/A	N/A	
				Human leukemia cell line JURKAT	✓	✗	89% growth inhibition at 2 mg/mL	N/A	N/A	
				Human leukemia cell line KASUMI-1	✓	✗	73% growth inhibition at 2 mg/mL	N/A	N/A	
				Human leukemia cell line KG-1	✓	✗	72% growth inhibition at 2 mg/mL	N/A	N/A	
				Human leukemia cell line LOUCY	✓	✗	45% growth inhibition at 2 mg/mL	N/A	N/A	
				Human leukemia cell line ML-2	✓	✗	69% growth inhibition at 2 mg/mL	N/A	N/A	[83]
				Human leukemia cell line MOLT-16	✓	✗	87% growth inhibition at 2 mg/mL	N/A	N/A	
				Human leukemia cell line MONO-MAC-6	✓	✗	65% growth inhibition at 2 mg/mL	N/A	N/A	
				Human leukemia cell line NB-4	✓	✗	75% growth inhibition at 2 mg/mL	N/A	N/A	
				Human leukemia cell line PEER	✓	✗	46% growth inhibition at 2 mg/mL	N/A	N/A	
				Human leukemia cell line U-266	✓	✗	43% growth inhibition at 2 mg/mL	N/A	N/A	
Bistramide A	Tunicate	*Lissoclinum bistratum*	Cyclic polyether	Human oralepidermoid carcinoma cell line KB	✓	✗	45 nM	N/A	N/A	[84]
				Murine leukemia cell line P388	✓	✗	20 nM	N/A	N/A	
Mandelalide B and E	Tunicate	*Lissoclinum sp.*	Polyketide	Human non-small cell lung cancer cell line NCI-H460	✓	✗	B: 25 nM, E: 2 µM	N/A	N/A	[85]
				HeLa cells	✓	✗	B: 23 nM, E: 1.9 µM	N/A	N/A	
				Human glioblastoma cell line U87-MG	✓	✗	B: 61 nM, E: >3 µM	N/A	N/A	
				Human colon carcinoma cell line HCT-116	✓	✗	B: 54 nM, E: >3 µM	N/A	N/A	

**Table 6:** List of promising anticancer marine products from mollusks studied in pre-clinical studies and reviewed in this work.

Compound Name	Marine Organism	Species Name	Active Derivative	Cancer Model	I n	I n	IC50 In Vitro	Route of Administration In Vivo	Dose Used In Vivo	Ref.
Jorumycin	Mollusk	*Jorunna funebris*	Isoquinoline alkaloid	Murine leukemia cell line P388	✓	✗	12.5 ng/mL	N/A	N/A	[86]
				Human lung adenocarcinoma cell line A549	✓	✗	12.5 ng/mL	N/A	N/A	
				Human colon cancer cell line HT29	✓	✗	12.5 ng/mL	N/A	N/A	
				Human melanoma cell line MEL28	✓	✗	12.5 ng/mL	N/A	N/A	
				Human colon cancer cell line HCT116	✓	✗	0.57 nM	N/A	N/A	[87]
				Human lung cancer cell line QC56	✓	✗	0.76 nM	N/A	N/A	
				Human prostate cancer cell line DU145	✓	✗	0.49 nM	N/A	N/A	
Dolastatin 10	Mollusk	*Dolabella auricularia*	Linear pentapeptide	Murine leukemia cell line P338	✓	✗	4.6 × 10^−5^ ng/mL	N/A	N/A	[88]
				Human ovarian cancer cell line OVCAR-3	✓	✗	9.5 × 10^−7^ µg/mL	N/A	N/A	
				Human glioma cell line SF-295	✓	✗	7.6 × 10^−6^ µg/mL	N/A	N/A	
				Human kidney cancer cell line A498	✓	✗	2.6 × 10^−5^ µg/mL	N/A	N/A	
				Human non-small cell lung cancer cell line NCI-H460	✓	✗	3.4 × 10^−6^ µg/mL	N/A	N/A	
				Human colon cancer cell line KM20L2	✓	✗	4.7 × 10^−6^ µg/mL	N/A	N/A	
				Human melanoma cell line SK-MEL-5	✓	✗	7.4 × 10^−6^ µg/mL	N/A	N/A	
				Human colon adenocarcinoma cell line LoVo	✓	✗	0.052 nM	N/A	N/A	[16]
				HeLa-derived cell line KB	✓	✗	0.076 nM	N/A	N/A	
Dolastatin 15	Mollusk	*Dolabella auricularia*	Linear pentapeptide	Human multiple myeloma cell lines RPMI8226, U266, and IM9	✓	✗	0.5–1 nM	N/A	N/A	[89]
Kahalalide F	Mollusk	*Elysia rufescens*	Depsipeptide	Human breast cancer cell line H5578T and Hs-578T	✓	✗	0.162 and 0.479 µM	N/A	N/A	[90]
				Human lung adenocarcinoma cell line A549	✓	✗	0.135 µM	N/A	N/A	
				Human colon cancer cell line (not specified)	✓	✗	0.162–0.288 µM	N/A	N/A	
Elisidepsin (KF synthetic derivative)	Mollusk	*Elysia rufescens*	Depsipeptide	Human breast cancer cell lines ZR-75-1, SKBR3, MDA-MB-361, MDA- MB-231, and MCF7	✓	✗	0.40, 0.5, 1.25, 4.7, and 8 µM, respectively	N/A	N/A	[91]
				Human colon cancer cell lines Colo205, HCC2998, HT29, Colo205R, and HCT116	✓	✗	0.75, 1.2, 3.7, 6.1, and 7.2 µM, respectively	N/A	N/A	
				Human head and neck cancer cell lines SQ20B, HEP2, and SCC61	✓	✗	3.5, 4.3, and 5.6 µM, respectively	N/A	N/A	
				Human hepatocarcinoma cell line SK-HEP1	✓	✗	6 µM	N/A	N/A	
				Human lung cancer cell lines HOP62 and HOP92	✓	✗	6.3 and 8 µM	N/A	N/A	
				Human melanoma cell line MDA-MB-435	✓	✗	4.4 µM	N/A	N/A	
				Human ovarian cancer cell lines IGROV1 and OVCAR3	✓	✗	4.2 and 7.3 µM	N/A	N/A	
				Human pancreatic cancer cell lines CAPAN1 and MiaCaPa2	✓	✗	5 and 8.8 µM	N/A	N/A	
				Human prostate cancer cell lines DU145 and PC3	✓	✗	1.26 and 1.8 µM	N/A	N/A	
Kulokekahilide-2	Mollusk	*Philinopsis speciosa*	Depsipeptide	Murine leukemia cell lines P338	✓	✗	4.2 nM	N/A	N/A	[92]
				Human ovarian cancer cell line SK-OV-3	✓	✗	7.5 nM	N/A	N/A	
				Human melanoma cell line MDA-MB-435	✓	✗	14.6 nM	N/A	N/A	

**Table 7:** List of promising anticancer marine products from bryozoans studied in pre-clinical studies and reviewed in this work.

Compound Name	Marine Organism	Species Name	Active Derivative	Cancer Model	In Vitro	In Vivo	IC50 in Vitro	Route of Administration In Vivo	Dose Used In Vivo	Ref.
Tambjamine K	Bryozoan	*Virididentula dentate*	Bipyrrolic alkaloid	Human colon cancer cell line HCT116	✓	✗	Cp 12: 13.7 µM, Cp 13: 3.6 µM, Cp 14: 0.14 µM	N/A	N/A	[93]
				Human breast cancer cell line MDA-MB-231	✓	✗	Cp 12: 15.3 µM, Cp 13: 3.5 µM	N/A	N/A	
Indole-based Tambjamine analogs	Bryozoan	*Virididentula dentate*.	Alkaloid	Human lung adenocarcinoma cell line A549	✓	✗	Cp 1: 10.66 µM and Cp 2: 7.61 µM	N/A	N/A	[94]
				Human small cell lung carcinoma cell line DMS53 and xenograft	✓	✓	Cp 1: 8.04 µM and Cp 2: 6.46 µM	i.p.	6 mg/Kg	
				Human lung squamous carcinoma cell line SW900	✓	✗	Cp 1: 8.67 µM and Cp 2: 7.55 µM	N/A	N/A	
				Human large cell lung cancer cell line H460	✓	✗	Cp 1: 8.37 µM and Cp 2: 7.29 µM	N/A	N/A	
				Human lung cancer primary culture #8	✓	✗	Cp 1: 4.04 µM and Cp 2: 3.34 µM	N/A	N/A	
				Human lung cancer primary culture #13	✓	✗	Cp 1: 4.34 µM and Cp 2: 4.03 µM	N/A	N/A	
Bryostatin 1	Bryozoan	*Bugula neritina*	Macrocyclic lactone	Murine leukemia cell line P388	✓	✗	0.25 nM	N/A	N/A	[95]
Bryostatin 5 and 8	Bryozoan	*Bugula neritina*.	Macrocyclic lactone	Murine melanoma K1735-M2 allograft	✗	✓	N/A	i.p.	1 µg	[96]

Abbreviations: N/A: Not applicable; i.v.: Intravenously; i.g.: Intragastrically; i.p.: Intraperitoneally; s.c.: Subcutaneously; PDX: Personalized derived xenograft; Cp: Compound; Ref.: Reference.

**Table 8:** Marine compounds approved and included in on-going phase III clinical trials for cancer treatment.

Compound Name	Commercial Name	Marine Organism	Active Derivative	Molecular Target	Cancer Type	Year of 1st Approval and Agency or Clinical Phase
Cytarabine (Ara-C)	Cytosar-U^®^ Depocyt^®^	Sponge	Nucleoside	DNA polymerase	Acute myeloid leukemia, non-Hodgkin’s lymphoma	196 9FDA
Fludarabine	Fludara^®^	Sponge	Nucleoside	DNA polymerase	Chronic lymphocytic leukemia, and indolent B-cell lymphoma	2008 FDA
Nelarabine (506U78)	Arranon^®^	Sponge	Nucleoside	DNA polymerase	T-cell acute lymphoblastic leukemia and T-cell lymphoblastic lymphoma	2005 FDA
Trabectedin (ET-743)	Yondelis^®^	Tunicate	Alkaloid	Minor groove of DNA	Soft tissue sarcoma, ovarian cancer	2007 EMEA
Eribulin mesylate (E7389)	Halaven^®^	Sponge	Polyketide	Microtubule	Locally advanced or metastatic breast cancer	2010 FDA
Brentuximab vedotin (SGN-35)	Adcetris^®^	Mollusk and cyanobacteria	ADC (anti CD30-MMAE)	CD30 and microtubules	Anaplastic large T-cell malignant lymphoma, Hodgkin’s lymphoma	2011 FDA
Plitidepsin	Aplidin^®^	Tunicate	Cyclic depsi-peptide	Rac1 and JNK activation	Multiple myeloma, T-cell lymphoma, leukemia	2018 ATGA
Polatuzumab vedotin (DCDS-4501A)	Polivy^®^	Mollusk and cyanobacteria	ADC (anti CD79b-MMAE)	CD79b and microtubules	Diffuse large B-cell lymphoma	201 9FDA
Plinabulin (NPI-2358)	NA	Fungi	Amide	Microtubules and JNK	Non-small cell lung cancer	III
Lurbinectedin (PM01183)	NA	Synthetic form from tunicate	Alkaloid	Minor groove of DNA	Small cell lung cancer Ovarian cancer	III
Depatuxizumab mafodotin (ABT-414)	NA	Mollusk and cyanobacteria	ADC (anti EGFR-MMAF)	EGFR and microtubule	Glioblastoma multiforme	III
Enfortumab vedotin (ASG-22ME)	NA	Mollusk and cyanobacteria	ADC (anti Nectin-4-MMAE)	Nectin-4 and microtubule	Urothelial cancer	III
Marizomib (NPI-0052)	NA	Bacteria	Beta-lactone	20S proteasome	Glioblastoma	III

Abbreviations: NA, not available; ADC, antibody drug conjugate; MMA, monomethyl auristatin.

**Table 9:** Limitations of the use of marine-derived drugs and strategies to overcome their limitations.

Limitations	Strategies to Overcome limitations	References
Lack of sustainable supply	Increase development of synthetic or hemi-synthetic derivatives from the biological source	[108,169]
Low production of bioactive compounds	Changing culture conditions, genetic engineering of organisms	[37,178,185]
	Properly designed and implemented extraction methodologies	[179]
Structural complexity of the marine compounds	In silico screening programs, NMR and MS	[75,185,186,187,188]
Correct taxonomic determination	Genomic approaches	[191,192,193,194]
Moderated efficacy	Conjugation with antibodies	[196]
	Encapsulation with nanoparticles	[201,202]
	Combination with other drugs	[201,202]
	Use of cell-penetrating peptides and tumor homing peptides	[197,199,200,203]
High market value	Rigorous planning on the usage of marine-derived drugs	[162]
